# Weak rTMS-induced electric fields produce neural entrainment in humans

**DOI:** 10.1038/s41598-020-68687-8

**Published:** 2020-07-20

**Authors:** Elina Zmeykina, Matthias Mittner, Walter Paulus, Zsolt Turi

**Affiliations:** 1 Department of Clinical Neurophysiology, University Medical Center Goettingen, Göttingen, Germany; 2 Department of Psychology, UiT – The Arctic University of Norway, Tromsø, Norway; 3 Department of Neuroanatomy, Institute of Anatomy and Cell Biology, University of Freiburg, Freiburg, Germany

**Keywords:** Neuroscience, Electroencephalography - EEG, Transcranial magnetic stimulation

## Abstract

Repetitive transcranial magnetic stimulation (rTMS) is a potent tool for modulating endogenous oscillations in humans. The current standard method for rTMS defines the stimulation intensity based on the evoked liminal response in the visual or motor system (e.g., resting motor threshold). The key limitation of the current approach is that the magnitude of the resulting electric field remains elusive. A better characterization of the electric field strength induced by a given rTMS protocol is necessary in order to improve the understanding of the neural mechanisms of rTMS. In this study we used a novel approach, in which individualized prospective computational modeling of the induced electric field guided the choice of stimulation intensity. We consistently found that rhythmic rTMS protocols increased neural synchronization in the posterior alpha frequency band when measured simultaneously with scalp electroencephalography. We observed this effect already at electric field strengths of roughly half the lowest conventional field strength, which is 80% of the resting motor threshold. We conclude that rTMS can induce immediate electrophysiological effects at much weaker electric field strengths than previously thought.

## Introduction

Neurons and neural assemblies in the mammalian brain temporally synchronize their activity leading to the emergence of macroscopic network oscillations^1^. Network oscillations are rhythmic patterns of neural activity that are maintained in all physiologically occurring brain states^2^. They are crucial for intact neuropsychological functioning and are frequently disrupted in neurological or psychiatric diseases^3^.

However, neurons also respond to both endogenous and exogenous electric fields^4^. Non-invasive electrical brain stimulation (NIBS) methods, such as repetitive transcranial magnetic stimulation (rTMS), are promising techniques for modulating endogenous oscillations^5^. Many NIBS studies employ oscillating electric fields because it is believed that these exogenous oscillations can modulate the phase or the power of endogenous oscillations^6^.

The two crucial properties of rTMS-generated periodic electric fields are its frequency and its magnitude. Whereas the frequency of the electric field is clearly defined, its magnitude in the brain is defined only indirectly. Most studies choose to set the stimulation intensity using the near threshold approach. This approach defines the stimulation intensity as a percentage of the threshold intensity required to induce a liminal response in the motor or visual cortex^7^.

Although the near threshold approach utilizes individualized stimulation intensities, the properties of the rTMS-induced electric field, including its strength, can differ substantially within and across individuals. For example, this approach cannot account for differences in the cortical folding pattern and the cortex-scalp distance between motor and non-motor areas^8^. However, it is crucial to account for these known anatomical effects because the induced electric field strength plays an important role in inducing electrophysiological effects^9^.

The induced electric field strength, however, remains unknown in most rTMS studies. A very limited number of retrospective estimations indicate that rTMS with conventionally used protocols induces peak electric field strengths of around 100 mV/mm^10,11^. At these high field strengths electrophysiological effects are consistently found^12–15^. However, these findings do not preclude the possibility that the effective threshold for rTMS is much lower. At least two separate lines of evidence support this assumption.

First, in vivo animal studies have shown weak, but reliable, electrophysiological effects already at field strengths in the range of 0.3 and 1 mV/mm^16,17^. This electric field range can temporally bias spike timing or might even entrain network oscillations^9,16^. Second, it has been found that even the weak electric fields induced by sham rTMS (ca. 5 mV/mm; 15-fold weaker than active rTMS) can induce short-lasting electrophysiological aftereffects in humans^10^.

Based on this converging evidence, we hypothesized that we should be able to observe immediate electrophysiological effects using electric fields between 20 and 50 mV/mm. The 20 mV/mm electric field corresponds to the stimulator’s lower limit of producing real rTMS (detailed in Supplemental Method, Validation measurement). This electric field range covers a “middle ground” between electrical brain stimulations, such as transcranial alternating current stimulation (tACS) and rTMS. On the one hand, the field strength is above the range of 0.3–1 mV/mm, and therefore stronger than the electric field used by conventional tACS^17^. On the other hand, these values are several orders of magnitude weaker than those used in the near threshold approach, where the electric fields are around 100 mV/mm^10^.

**Figure 1:**
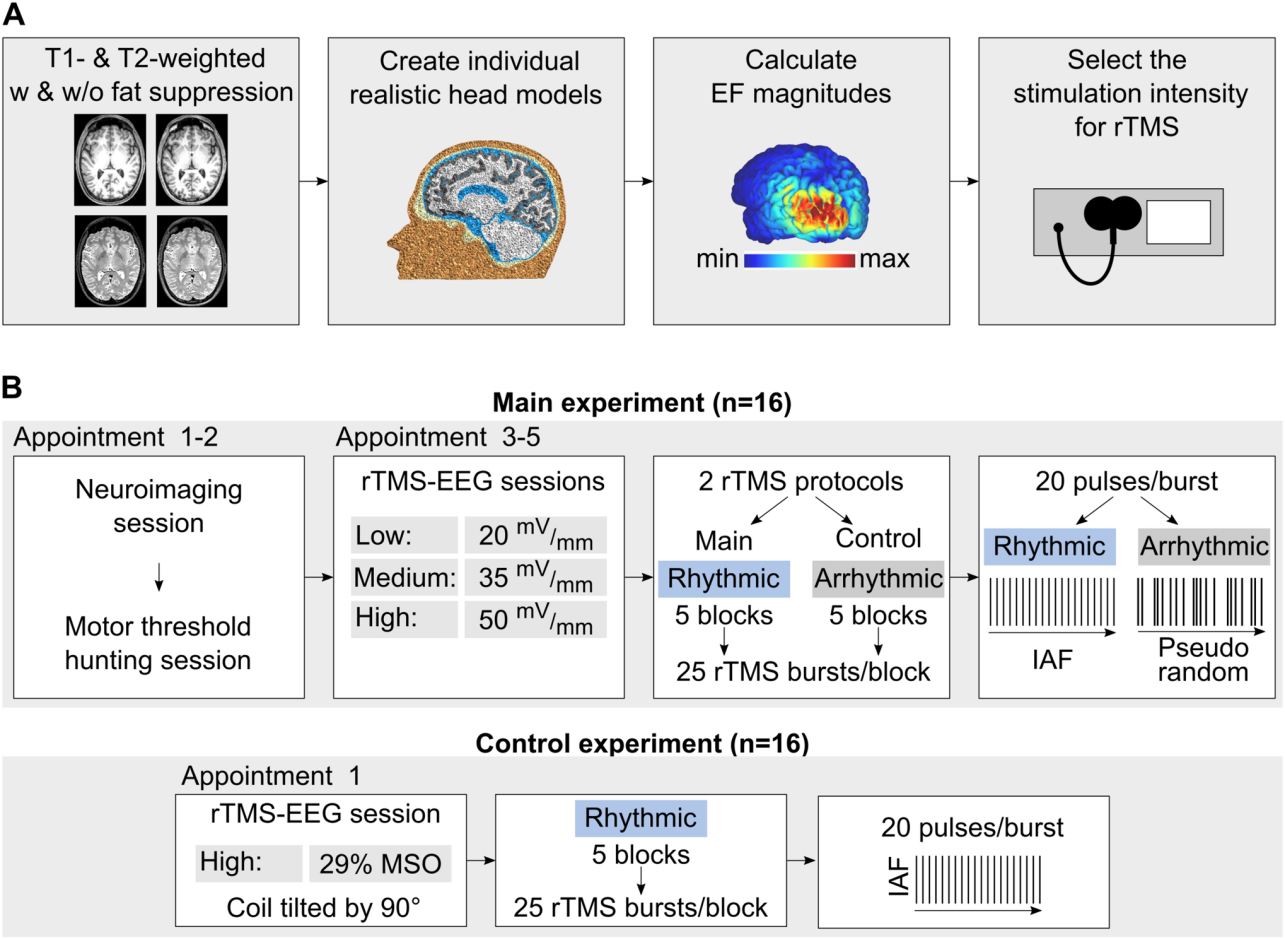
Study overview. (**A**) Schematic of the prospective electric field estimation approach. (**B**) Appointments and stimulation protocols in the main (top) and control (bottom) experiments. *EF* electric field, *MSO%* percentage of maximum stimulator output, *IAF* individual alpha frequency.

To test our hypothesis, we took an alternative approach to the conventional near threshold method. We refer to it as the prospective electric field estimation approach (for an overview see Fig. 1A and Table S1). The decisive feature of our approach is that prospective computational modeling of the magnitudes of the induced electric fields guided the choice of stimulation intensity at the individual subject level. Moreover, we estimated individual peak frequencies of posterior alpha oscillations to fine-tune the stimulation frequency. Finally, real-time neuronavigation ensured accurate and consistent targeting across the sessions.

By using this approach, our focus was on inducing immediate electrophysiological effects on posterior alpha oscillations in humans. The reasons for focusing on posterior alpha oscillation are that it has a high signal-to-noise ratio in resting state measurements, and that its peak frequency has a low intra-subject variability^18^. To characterize the immediate electrophysiological effects of rTMS, we calculated the phase locking value (PLV) of the simultaneously recorded scalp electroencephalography (EEG)^15,19,20^. The PLV captures the extent of neural synchronization by measuring the amount of phase alignment between the rTMS and network oscillations assessed by EEG. Our novel individualized intensity selection method for rTMS with prospective electric field modeling shows that neural entrainment occurs at lower than expected field strengths.

## Results

### Study overview

The present study consisted of a main and a control experiment (for an overview see Fig. 1). In the main experiment (Fig. 1B, top), we employed a single-blind, randomized, cross-over study design, using an active control rTMS condition within participants and sessions. The participants (n = 16) took part in five experimental appointments including one neuroimaging session, one session for motor threshold hunting, and three rTMS-EEG sessions.

In the rTMS-EEG sessions, the participants received rTMS stimulation at intensities prospectively estimated to induce EFs of three different magnitudes: 20, 35 and 50 mV/mm. These values correspond to the group-level mean (± SD) of 9.5 ± 1.1%, 16.8 ± 2% and 23.9 ± 2.5% of the maximum stimulator output, respectively. We obtained peak magnitudes of the absolute electric field extracted from the gray matter compartment. The center of the coil was placed over the PO3 electrode. We applied each dose on individual sessions separate by at least 48 h. During each rTMS-EEG session, we applied rhythmic (main) and arrhythmic (active control) rTMS protocols. All stimulation parameters except the rhythmicity were identical in both protocols.

In the rhythmic protocol, we set the stimulation frequency to the individual alpha frequency following the Arnold tongue model. This model assumes that neural entrainment is most effective when the stimulation frequency matches the endogenous frequency^12,21^. In the arrhythmic protocol, the same number of pulses as in the rhythmic protocol was presented but the inter-pulse interval was randomized in order to remove frequency-specific stimulation effects^12,15^. Apart from the rhythmicity, all stimulation parameters (stimulation intensity, location, number of TMS pulses) were identical in both protocols. The two protocols would probably produce closely matched acoustic and somatic sensations. We applied the stimulation with the participants at rest, and we instructed them to keep their eyes open.

In order to control for potential effects induced by the acoustic by-products of the rTMS device, we performed an additional control experiment on a separate group of participants (n = 16; Fig. 1B, bottom). We chose a commonly used sham procedure, in which we tilted the stimulation coil by 90° (e.g.,^14^). This sham protocol emulates the rTMS-induced click sounds that might induce a spurious increase in occipital alpha synchronization^22^ while minimizing any direct effects of the stimulation. In the control experiment, the participants received a single rhythmic rTMS session applied at a fixed stimulation intensity of 29% of the maximum device output. This value corresponds to the highest stimulation intensity applied in the main experiment. We chose this value in order to maximize the noise level of the sham rTMS, which increases with stimulation intensity. The stimulation frequency was set to the individual alpha frequency. Apart from the stimulation intensity, all the remaining parameters were kept constant as in the main experiment (detailed in “Simultaneous rTMS and EEG” section).

### Rhythmic rTMS synchronizes ongoing posterior alpha rhythms

We performed the following analysis to characterize the immediate electrophysiological effects of rTMS in the main and control experiment. In the preprocessing stage of the data analysis, we removed the TMS-induced artifacts from the EEG data. We used the same preprocessing algorithm for all stimulation conditions. A detailed description is given in “Analysis of rTMS-EEG” section. In brief, we eliminated ringing artifacts by removing data from 4 ms before to 9 ms after the TMS pulse. Next, we ran an independent component analysis (ICA) to remove decay artifacts. We then interpolated the time interval around each TMS pulse.

**Figure 2:**
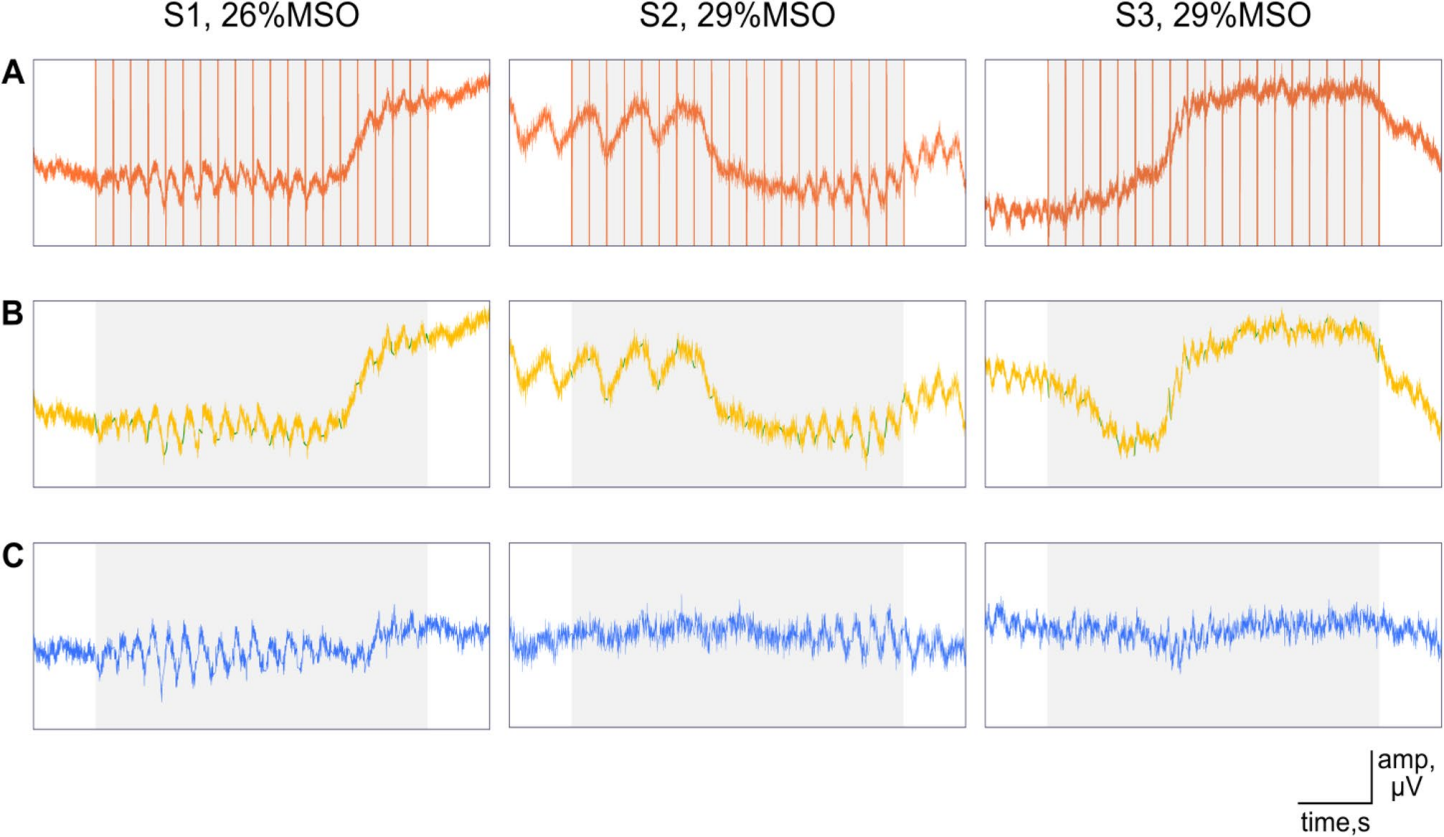
Artifact removal for three example datasets. Onset and offset of rTMS are shown as grey rectangle, individual pulses are shown by red vertical lines. (**A**) Raw data from the POz channel. (**B**) The removal and interpolation (green) of ringing artifacts. (**C**) Data at the end of preprocessing.

Further, we used a semi-automatic algorithm adapted from the open-source toolbox ARTIST to eliminate further artifacts^23^. We defined trials or channels as contaminated with artifacts if their power exceeded 1.5 times the interquartile range. If the artifacts affected fewer than 20% of all channels, we interpolated signals from the non-contaminated channels, or otherwise removed the entire trial. Moreover, we removed channels with a large standard deviation (STD > 30 µV). We also estimated the correlation coefficient of the signal of each channel with its neighbors and removed and interpolated those channels with a low correlation coefficient (< 0.4). Finally, we removed blinks, saccades and other eye-related movements by ICA. Figure 2 shows the raw data before and after the artifact removal process from three example participants.

Next, we measured the amount of synchronization in the scalp EEG signal. For this, we first applied wavelet decomposition of the EEG signal and extracted the phase information from the imaginary component of the Fourier coefficient (see “Analysis of rTMS-EEG” section). We then simulated sinusoidal waves based on the individual stimulation frequencies and phase-aligned them to the offset of the TMS pulses. We computed the PLV between the EEG signal and the sinusoidal wave for each rTMS intensity condition and rTMS protocol, respectively.

**Figure 3:**
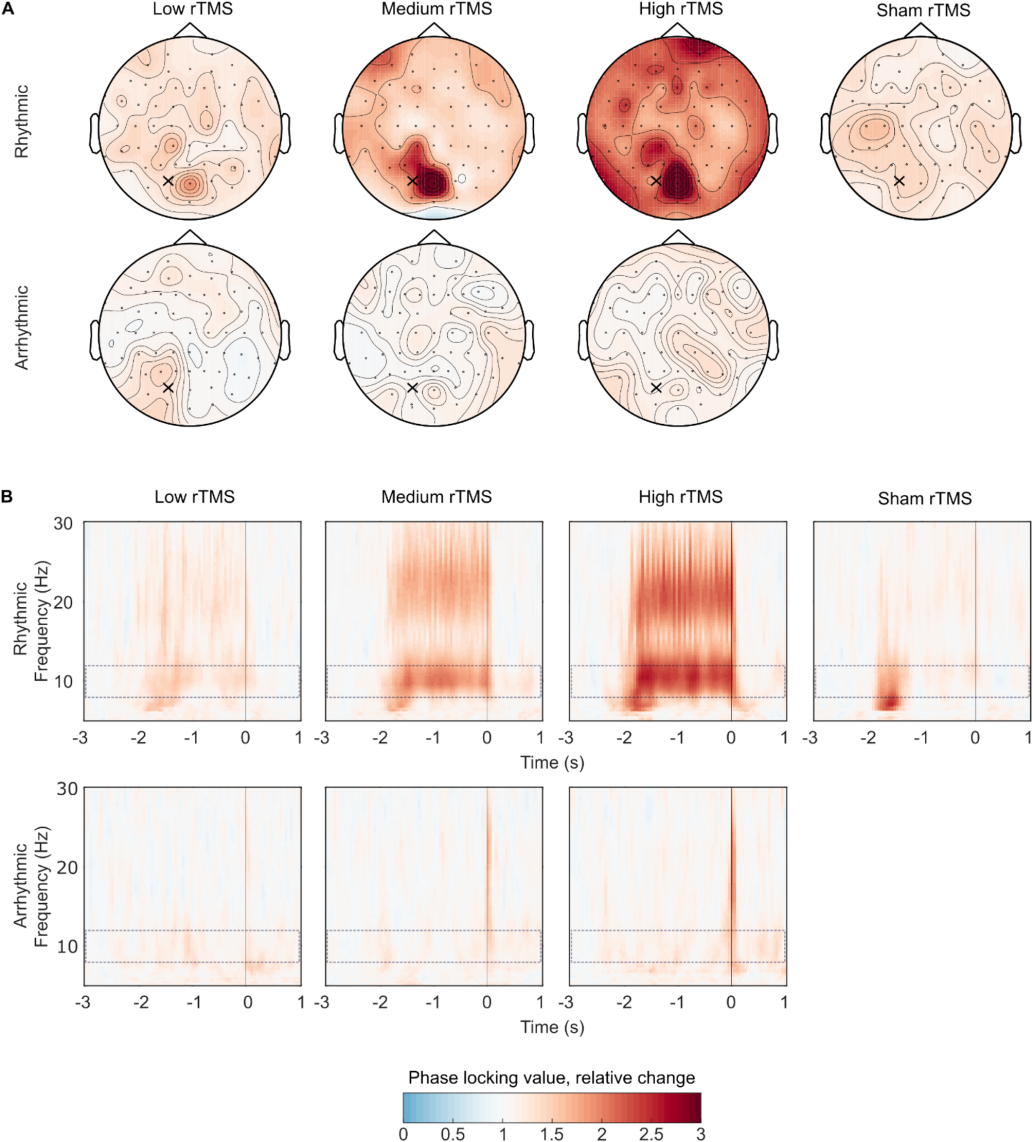
Rhythmic rTMS synchronized ongoing posterior alpha rhythms indicated by increased phase locking values. (**A**) The degree of synchronization at the individual alpha frequencies was most pronounced in the posterior electrodes for the real rhythmic (top row) but not for the sham rhythmic (top, right) or arrhythmic rTMS (bottom row). We applied rTMS over the PO3 electrode (location marked with the cross). (**B**) Rhythmic rTMS (top row) synchronized ongoing posterior alpha rhythms and its first harmonics in the posterior electrodes. Compared to baseline, sham rTMS induced a short-lasting increase in the PLVs in the theta and alpha frequency bands. The alpha band is shown with a dashed rectangle. Color represents the changes of phase locking value relative to baseline from − 3 to − 2.5 s prior to rTMS offset. Timepoint t = 0 corresponds to the last pulse of all rTMS bursts in (**B**).

Based on the Arnold tongue model of neural entrainment^21^, we expected that the intervention would synchronize the ongoing endogenous brain rhythm to the rhythmic rTMS. We also expected that the arrhythmic or sham protocols would not affect the amount of synchronization. To test this hypothesis, we first determined how rTMS affected the amount of neural synchronization as measured by the PLV in the individual alpha frequency relative to baseline. We defined the baseline as the time window 500 ms before rTMS onset. We normalized to baseline with the relative-change method: A baseline normalized value of 1 indicates no change in the PLV, a value of 0.5 shows a 50% decrease and value of + 1.5 corresponds to a 50% increase. We found that rhythmic rTMS increased the PLV across all rTMS intensities (Fig. 3A, top), while arrhythmic rTMS had no such effect (Fig. 3A, bottom). As expected, with rhythmic rTMS we observed the greatest PLV increase over the posterior electrodes (Fig. 3A, top). This was not the case for the arrhythmic or sham protocols.

Because we stimulated the posterior parietal-occipital cortex, we studied the time course of PLV change in the posterior electrodes for frequencies between 5 and 30 Hz (Fig. 3B). We aligned the data to the offset of the rTMS burst, which is indicated by the vertical line at (0 s) on the time axis. Because we delivered rTMS at the individual alpha frequency and kept the number of pulses constant, the duration and hence the onset time of the rTMS bursts varied (e.g., 8 Hz: 2.5 s; 12 Hz: 1.67 s). In the rhythmic rTMS protocol, we found that PLV increased after rTMS onset and returned to the baseline after rTMS offset (Fig. 3B, top). The increase in PLV was strongest in the ongoing alpha frequency band and its harmonics in the beta frequency range. For the arrhythmic protocols, we found no change in the PLVs (Fig. 3B, bottom). For the sham rTMS, we observed an initial increase in the theta and alpha frequency bands (Fig. 3B, right) but no sustained increase in PLV throughout stimulation.

**Figure 4:**
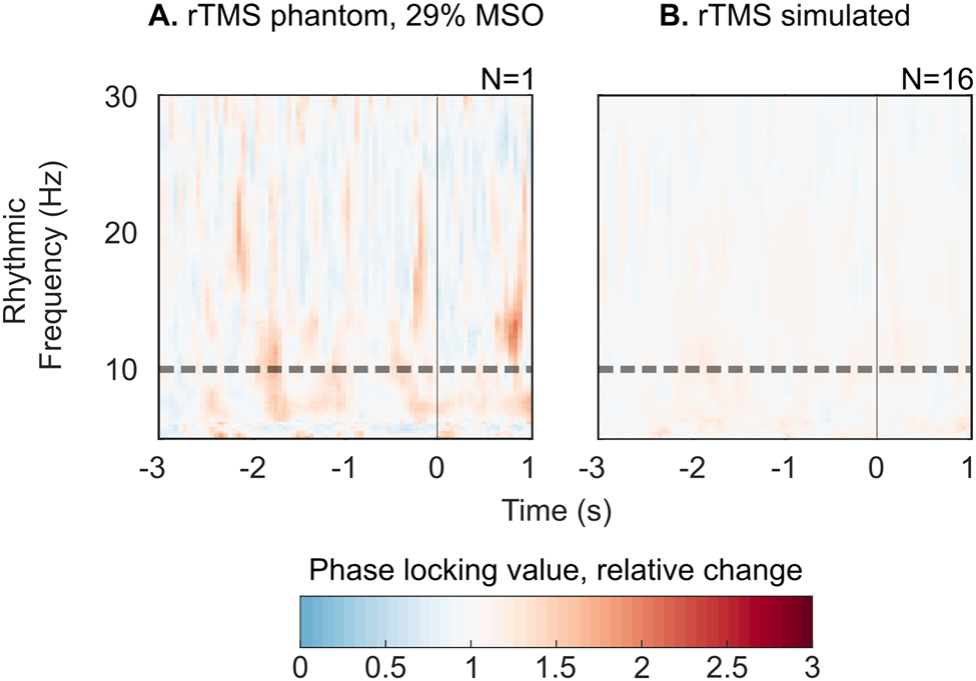
Preprocessing pipeline does not artificially increase the degree of neural synchronization. (**A**) Control measurement on piece of meat. (**B**) Control analysis on artifacts free resting state EEG data.

We performed two control analyses to ensure that the PLVs change in the main rhythmic rTMS condition was not due to artifacts or induced by our preprocessing pipeline (detailed in “Analysis of rTMS-EEG” section). First, a spurious increase in PLVs can potentially arise if ringing and decay artifacts are only incompletely removed. We therefore performed a control measurement on a piece of meat using the identical stimulation, measurement and analysis parameters as in the main experiment. This analysis confirmed that our preprocessing pipeline removed these artifacts, as we detected no increase in PLV (Fig. 4A).

Second, a potential bias could arise from the periodical exclusion of intervals corresponding to the rTMS-induced ringing artifacts and their interpolation. We tested for this bias by performing a control analysis on artifact-free resting state EEG data that had been recorded from the 16 participants before each rTMS-EEG session in the main experiment (see “Simultaneous rTMS and EEG” section). This control analysis confirmed that our analysis pipeline did not increase the PLVs under the main rhythmic rTMS condition (Fig. 4B).

### rTMS induces rapid and sustained increase in the ongoing posterior alpha synchronization

**Figure 5:**
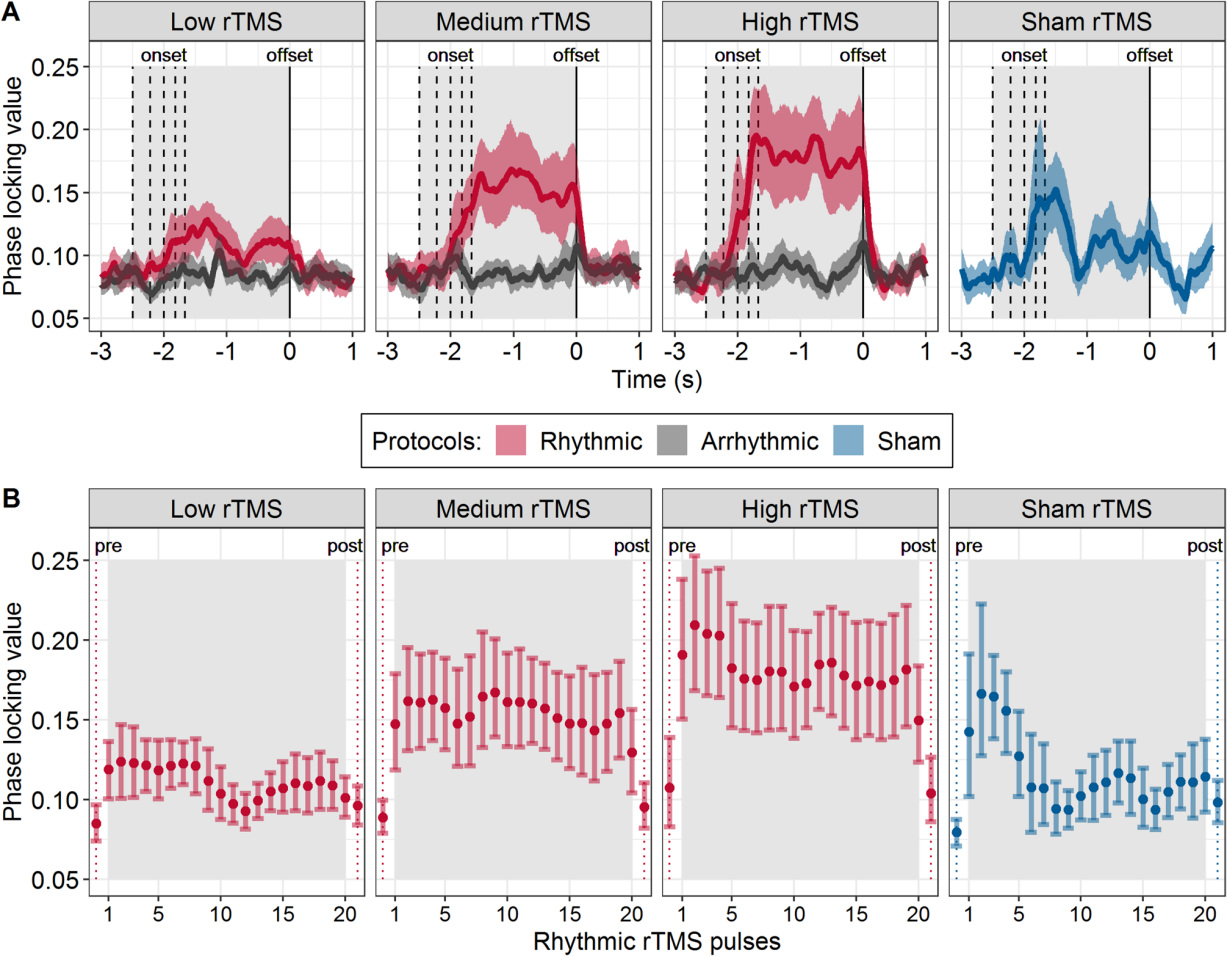
Increased and sustained neural synchronization during rhythmic but not during arrhythmic or sham rTMS. (**A**) The time course of the phase locking value calculated for individual alpha frequencies is shown for the parietal channels. Vertical dashed lines correspond to the stimulation onset at individual alpha frequencies (between 8 and 12 Hz; from left to right). The vertical solid line shows the rTMS burst offset (0 s). Phase locking values are aligned to the offset of the rTMS burst. Solid lines represent mean phase locking values, the shaded areas show the 95% confidence intervals. (**B**) Phase locking values for the real and sham rhythmic rTMS conditions, aligned to each of the 20 rTMS pulses. The dots represent means, and the error bars 95% confidence intervals. Dotted lines show phase locking values before the first rTMS pulse (pre) and after the last rTMS pulse (post). The light gray rectangle highlights the time window during which rTMS was applied.

Next, we focused on the PLV time course at the individual alpha frequency (Fig. 5). In the rhythmic rTMS condition, the PLV increased rapidly after the onset of rTMS and returned to baseline after stimulation offset (Fig. 5A). The offset-locked data analysis introduced variability in the initial part of the time course of the PLV, due to varying onsets of the stimulation for each participant. Thus, we also computed PLVs locked to each TMS pulse (Fig. 5B). This analysis was only conducted on the rhythmic rTMS protocols since they are characterized by constant inter-pulse intervals.

The initial pattern of PLV increase was similar for all rhythmic rTMS intensity conditions. However, the time course of the PLV change was slightly different for the three main rTMS intensity conditions. In the low rTMS intensity condition, we found that the mean PLVs returned to the baseline after an initial increase (Fig. 5B, left). In the middle and high rTMS intensity conditions, the induced increase in the PLV was stable over the time course of the stimulation (Fig. 5B, middle and right) and the level on which the PLV plateaued was higher for the highest intensity condition. We observed an initial increase in the PLVs also in the sham condition. However, this initial increase shortly returned to the baseline value after the first five pulses and did not show the sustained pattern observed in the two active stimulation conditions.

We then compared the PLVs during the stimulation period of the rhythmic, arrhythmic and sham rTMS protocols using independent non-parametric cluster-based permutation tests at each rTMS intensity condition. The PLVs in the rhythmic rTMS were significantly higher than in the arrhythmic rTMS at medium and high intensities (both p < 0.001) but just missed significance at the low rTMS intensity (p = 0.054). Sham rTMS resulted in significantly higher PLVs compared to the rhythmic low rTMS (p = 0.011) and arrhythmic rTMS at all intensities (p < 0.001). Real rhythmic rTMS applied at medium and high intensities resulted in significantly higher PLVs than the sham rTMS (p = 0.042 and p = 0.003).

### Mean and median electric field strengths are in the effective range

**Figure 6:**
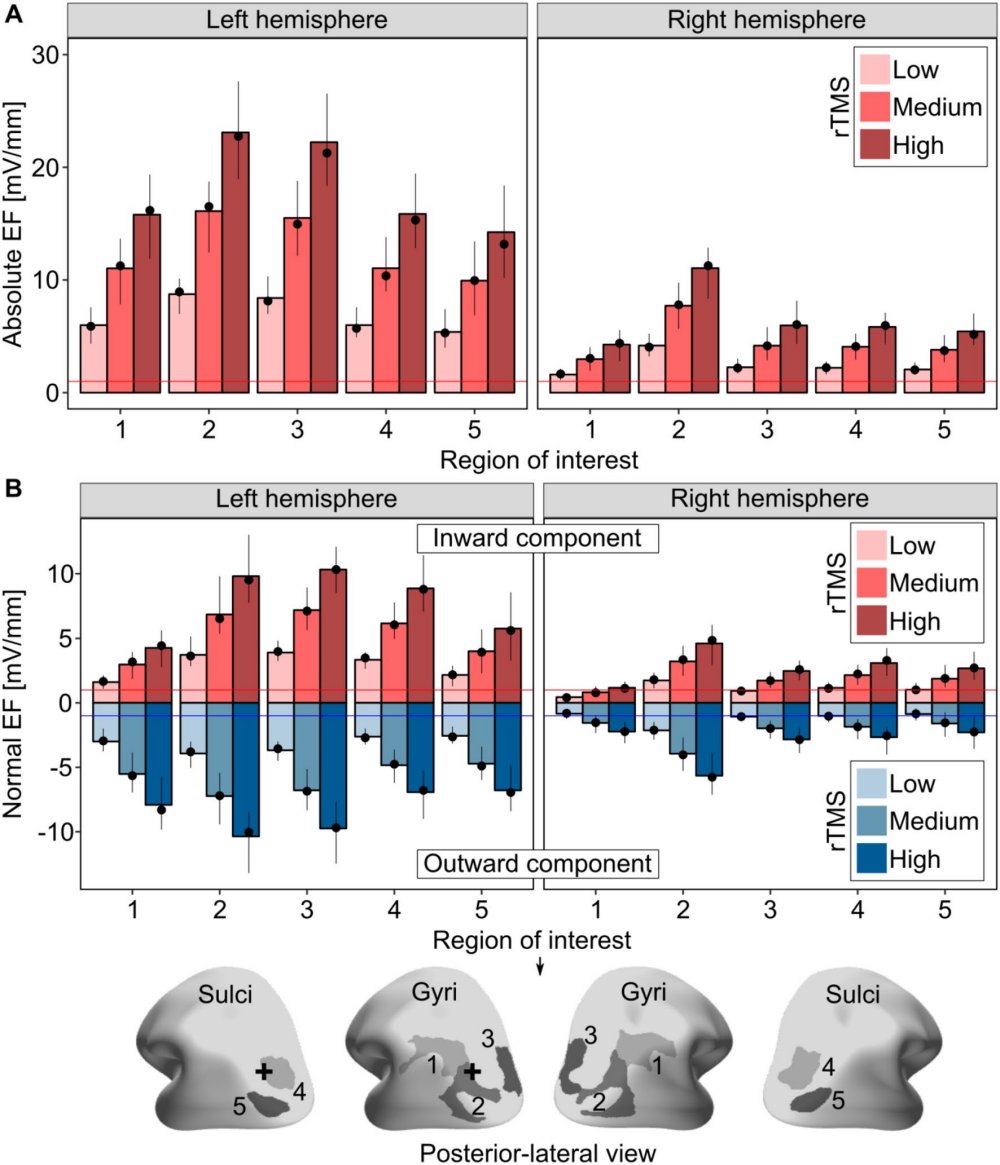
Group-level mean and median values of the electric field for parietal and occipital ROIs. Bar plots show the mean, dot plots show the median electric field values of the (**A**) absolute EF and (**B**) its normal component. Range plots correspond to the 2.5th and the 97.5th percentiles of the electric field values, respectively. Red and blue horizontal lines correspond to the 1 mV/mm and − 1 mV/mm EF strengths. A black plus sign indicates the center of the TMS coil. Range of interest: 1—Angular gyrus, 2—Superior occipital gyrus, 3—Middle occipital gyrus, 4—Superior occipital sulcus and transverse occipital sulcus, 5—Middle occipital sulcus and lunate sulcus.

We characterized the mean and median electric field (EF) strengths across a number of posterior regions of interest (ROIs) as shown in Fig. 6 and the peak electric field values shown in Figure S1. We selected regions in the parietal and occipital lobes that were in the vicinity of the stimulation target. As previous in vivo animal studies have demonstrated immediate electrophysiological effects at EF strengths of ca. 1 mV/mm (e.g.,^17^), we used this value as the reference to which we compared the rTMS-produced EF values observed in the present study.

For both the absolute EF (Fig. 6A) and for the normal component (Fig. 6B), the mean and median EF strengths were higher than the 1 mV/mm reference value (and weaker than − 1 mV/mm) in all rTMS conditions in the target hemisphere. In the right hemisphere, most ROIs experienced an EF stronger than 1 mV/mm only in the medium and high intensity conditions. Similarly, the peak EF values (both the absolute and the normal component) were higher than the 1 mV/mm reference value in both hemispheres (see Figure S1).

### Immediate effects in the range of 30 to 42% of the resting motor threshold

**Figure 7:**
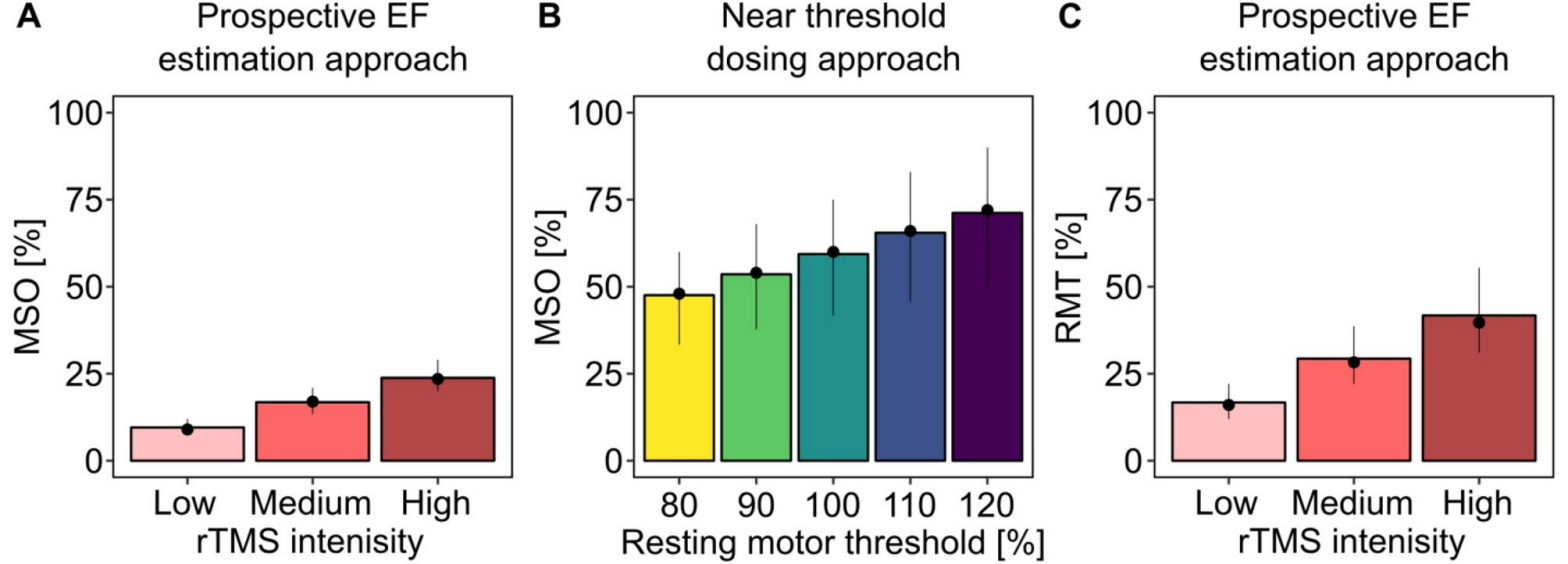
Our prospectively determined rTMS settings fell in the range of 17–42% of the resting motor threshold. (**A**) The resulting electric fields and (**B**) the resting motor thresholds between 80 and 120% expressed in percentages of the maximum stimulator output. (**C**) Resulting electric fields expressed in percent of the 100% resting motor threshold. Bar plots show means, and dots the median values. Range plots correspond to the 2.5th and the 97.5th percentiles, respectively. *EF* electric field, *MSO* maximum stimulator output, *RMT* resting motor threshold.

In order to make our results more interpretable, we expressed the EF strengths in terms of maximum stimulator output (Fig. 7A), and resting motor threshold percentages (Fig. 7C). We used the motor threshold approach because it is the most frequently used approach in the literature (Table S1). We also characterized the resting motor threshold as the percentage of the maximum stimulator output (Fig. 7B). Intensities in the range of ca. 30–42% of the resting motor threshold were already capable of inducing immediate electrophysiological network effects in humans. The low stimulation intensities were well tolerated by the participants, who reported no phosphenes, and only a minor amount of somatosensory discomfort during rTMS (see Figure S2), which is a major concern when using higher stimulation intensities (e.g.,^24^).

## Discussion

Using prospectively individualized intensities for rTMS we showed that electric fields half the magnitude of conventionally applied fields (see Figure S1B and D) already induced immediate electrophysiological effects in humans. In the rhythmic rTMS protocols, the amount of neural synchronization increased rapidly after rTMS onset and returned to the baseline after rTMS offset. The field strength played a modulating role in inducing the effects. In the low rTMS intensity condition, the immediate effects were not statistically different from sham rTMS. In the medium and high rTMS intensity conditions, the effect was statistically significant and appeared to be stable over the time course of the rTMS burst. This was not the case for the sham rTMS, which only produced an initial, short-lived effect. Furthermore, we observed different PLV topographies between the real and the sham rTMS protocols (Fig. 3A). In the real protocols, the PLV increase appeared over the middle parietal and occipital electrodes. In the sham protocol on the other hand, we observed the highest PLVs over the left temporal electrodes. In the arrhythmic protocol, which served as an active control, rTMS did not affect the amount of neural synchronization. We conclude that rhythmic rTMS applied at peak absolute electric fields from 35 to 50 mV/mm can induce immediate electrophysiological effects in humans.

### Comparing our results with previous electrophysiological findings

In the rhythmic rTMS protocol, the observed pattern in the time course of the neural synchronization reproduced many aspects of previous tACS and rTMS findings^12,14,16,17^. Animal studies assessing spike timing activity revealed an immediate increase in the degree of neural synchronization during tACS^16,17^. Similar to our own findings, this increased activity returned to baseline immediately after the end of stimulation^16,17^.

A previous study assessed the effect of rhythmic rTMS at the individual peak beta frequency^14^. The participants received rTMS with a conventional stimulation intensity (90% of active motor threshold) while at rest^14^. The authors found that rhythmic rTMS increased the degree of neural synchronization compared to sham stimulation (coil tilted by 90°) or to control frequencies^14^.

On the other hand, our findings are slightly different than those of another rTMS study^12^, in which the stimulation was applied at the individual peak alpha frequency over the parietal cortex with the conventional intensity of 100% phosphene threshold^12^. These authors found that after an initial rapid increase, the degree of neural synchronization gradually decreased during the second part of the rTMS burst^12^. Surprisingly, the arrhythmic protocol in their study also initially increased the degree of neural synchronization^12^. This is contrary to our findings as we failed to find any changes in neural synchronization in our arrhythmic protocol.

One possible explanation for these divergent findings might be that even single pulse high-intensity TMS can induce alpha frequency oscillations in the occipital cortex^25,26^. These studies used high TMS intensities of approx. 100 mV/mm, which could have induced the observed degree of neural synchronization with the first rTMS pulse in the arrhythmic protocol^12^. Careful analysis of our own data revealed a similar pattern, especially with the higher intensity conditions. There seems to be a slight increase in the degree of synchronization after the last rTMS pulse in the arrhythmic protocol (see Figs. 3B and 5A). Note that our analysis was locked to the offset rather than to the onset of the rTMS burst. However, maintaining the neural synchronization over time requires rhythmically delivered rTMS pulses**.**

### Relation of electric field strengths to previous tACS and rTMS studies

The electric field magnitudes in the medium and high rTMS conditions are several-fold stronger than the already effective magnitudes observed in animal studies^16,17^. Furthermore, the employed intensities were several-fold weaker than those applied in previous rTMS studies^12,15^ and therefore cover a “middle ground” between the two techniques that may be particularly suited to study immediate electrophysiological effects.

However, it is challenging to directly compare the exact electric field values in the literature, because systematic studies are lacking. Instead, studies are divided on species, stimulation frequencies, stimulation methods and the state of the receiving brain^16,17,27^. For example, the differences in the electric fields generated by tACS and rTMS make a direct comparison difficult. One important difference relates to the cycle/pulse duration. For tACS, depending on its frequency, a single cycle is in the range of up to several hundreds of ms. On the other hand, the width of a biphasic rTMS pulse lasts for only several hundred of µs. This difference is crucial because pulse duration is an important temporal characteristic of the induced field. Keeping the magnitude and the pulse/waveform constant, longer pulses deliver a higher total charge than shorter pulses^28^. Another important difference between tACS and rTMS are the spatial components (e.g., normal, tangential) of the induced electric field, with TMS inducing stronger tangential components than tACS^29^.

### Towards a better understanding of the neural mechanisms of rTMS

For several decades, the near threshold approach has been the most commonly used intensity selection method for rTMS^7,30^. This approach is uncomplicated and offers individualized stimulation intensities. It selects the stimulation intensity based on evoking a liminal response in the visual or motor system^31^ and therefore offers a rough approach to taking individual variation into account.

Externally inducing liminal responses, however, requires a strong electric field, and the functional effect of such an electric field is mostly interferential in nature. The prime example of such functional interference is rTMS-induced speech arrest^32^. When rTMS is applied over the left posterior-inferior frontal region, i.e., the facial motor cortex, it can block the ability to speak^32^.

Yet, the purpose of rTMS in neuroscience and clinical applications is primarily a targeted enhancement of function rather than interference (but see^33^). From a neuroscience point of view, achieving functional enhancement is more challenging than producing interference. This is because the former requires a better understanding of the involved neural mechanisms. But what neural mechanisms does rTMS activate when applied at 80 or 120% of the resting motor threshold? One crucial limitation of the near threshold method is that it offers no clear physiological justification for the choice of stimulation intensity; the magnitude of the resulting electric field as well as the underlying neural mechanism of rTMS remain unknown.

Recent developments in computational modeling may help to overcome this limitation as exemplified in the present study. Anatomically realistic head modeling and electric field calculations have the merit of linking the stimulation intensities to the resulting electric field strengths^34–36^. This can facilitate the mapping between electric field strength, neural mechanisms and functional effects^37^. A better understanding of the neural mechanisms of rTMS benefits both neuroscience research and the clinical application of the method^38,39^. For example, many such applications are aimed at restoring altered oscillatory activity, e.g. in schizophrenia, stroke or epilepsy^40^. One can achieve this by the targeted external control of altered oscillations though specific neural mechanisms, e.g. neural entrainment.

However, even the state-of-the-art head-modeling approaches are only an approximation of the true individual anatomy. Known sources of inaccuracy include segmentation errors^41^, a limited number of tissue types^42^, and the use of standard, but possibly incorrect, conductivity values^43,44^. Despite these limitations, we remain convinced that computational models are invaluable for prospectively adjusting the stimulation intensities for rTMS. They can provide new insights into how an electric field generated from outside the head by rTMS can produce immediate electrophysiological effects in the human brain. We conclude that individualized prospective electric field strength calculation is an essential approach to better understand the neural mechanisms of rTMS.

### Future directions

The precision of determining the anatomical target possibly further affects the immediate electrophysiological effects. We expect that individualizing the stimulation target based on anatomy, e.g., targeting the inferior parietal sulcus, or EEG source estimation-based localization would further increase the efficacy of rTMS. When using these ultra-low intensities, following the Arnold tongue demands to adjust stimulation frequency and location increase substantially. Otherwise, the intervention will likely to miss effects.

It is unclear whether the observed changes in neural synchronization would manifest in observable behavioral effects. A candidate mechanism through which such changes could manifest is increased cortical inhibition via increased neural synchrony. In general, at low intensities inhibitory circuits seem to be stimulated preferentially^45,46^. Also, alpha activity has been associated with cortical inhibition^47–49^, thus, hyperpolarization instead of excitation may play a decisive role.

## Methods

### Participants

We recruited neurologically healthy volunteers in this study (see Table 1). We included participants, if we could estimate the individual alpha frequency in the eyes closed or open resting state conditions. In the main experiment, the dataset of one was incomplete and was excluded from further analysis. We used the Edinburgh Handedness Inventory^50^ to estimate the laterality index of our participants. The sample size was determined based on earlier rTMS-EEG studies^12,14,15^.

**Table 1:** The participant information in the main and control experiments.

	Main experiment	Control experiment
Final sample size	16	16
Excluded participant(s)	1	0
Mean age ± SD (years)	25.5 ± 3.2	23.9 ± 3.9
Age range (years)	From 21 to 32	From 20 to 34
Number of women/men	8/8	8/8
Mean laterality index ± SD	78.4 ± 50.1	78.8 ± 31.6
Laterality index range	From -30 to 100	From 0 to 100

In the main experiment, the dataset of one participant was incomplete and was excluded from further analysis.

Before participation, all volunteers filled out self-completed questionnaires to assess the study exclusion criteria. In cases of possible contraindications, a neurologist at the Department Clinical Neurophysiology, University Medical Center Göttingen examined the volunteer. Inclusion criteria were no history or presence of medical, neurological or psychiatric illnesses including epilepsy, drug and/or alcohol abuse, and no metal implants in the head, neck, or chest.

### Ethic statement and research integrity

The Ethic Committee of the University Medical Center Göttingen approved the investigation, the experimental protocols, and all methods (Application number: 35/7/17). We performed all experiments in accordance with relevant guidelines and regulations. All participants gave written informed consent before participation. The raw data and code for the reported analyses are available for download at our repository (https://github.com/ZsoltTuri/2019_rTMS-EEG).

### Procedure

In the main experiment, the participants took part in one neuroimaging session were we collected anatomical, diffusion weighted and functional magnetic resonance imaging data. In the next session, we estimated the resting motor threshold by using neuronavigated single pulse TMS (spTMS). Preceding the three rTMS-EEG sessions, we performed the head modeling and EF calculations to prospectively estimate the stimulation intensity for each individual and session. In the control experiment, the participant took part in one rTMS-EEG session. Unknown to the participants, they received only sham rTMS.

### Acquisition and analysis of neuroimaging data

#### Acquisition

Magnetic resonance images (MRI) were acquired using a 3 T MRI-scanner (Siemens Magnetom TIM Trio, Siemens Healthcare, Erlangen, Germany) equipped with a 32-channel, commercial head coil (Siemens Healthcare, Erlangen, Germany). 3D T_1_-weighted datasets were obtained using Magnetization Prepared Rapid Gradient-Echo (MP-RAGE) acquisitions with or without selective water excitation for fat suppression employing the following parameters: Turbo fast low angle shot (Turbo FLASH), echo time (TE): 3.26 ms, repetition time (TR): 2,250 ms, inversion time: 900 ms, flip angle: 9°, receiver bandwidth: 200 Hz/Px that cover the whole head at 1 × 1 × 1 mm^3^ isotropic resolution.

3D T_2_-weighted Turbo spin echo (TurboSE) sequences were acquired with and without fat suppression using the following imaging parameters: TE: 282 ms, TR: 3,500 ms, slice number: 176, slice thickness: 1 mm, field of view (FoV; longitudinal coverage): 256 mm, echo spacing: 4.84 ms, turbo factor 125, receiver bandwidth: 355 Hz/Px that cover the whole head at 1 × 1 × 1 mm^3^ isotropic resolution.

For diffusion-weighted imaging, single-shot spin-echo echo planar imaging sequences were obtained using the following parameters: TE: 88 ms, TR: 10,000 ms, slice thickness: 1.7 mm, FoV longitudinal coverage: 218 mm, receiver bandwidth: 1,346 Hz/Px. For accelerating T_2_- and diffusion-weighted image acquisitions, we obtained parallel imaging techniques by means of generalized autocalibrating partially parallel acquisitions (GRAPPA) with a twofold acceleration factor.

In addition, the participant performed rhythmic, stereotypic movements with the first dorsal interosseous muscle, i.e. thumb adduction, to localize its cortical representation. We used a gradient-echo planar imaging (EPI) sequence to detect BOLD changes by using the following imaging parameters: TR/TE: 900 ms/30 ms, flip angle = 50°, voxel size 3 × 3 × 3 mm^3^, field of view (FOV) 210 × 210 mm, 39 slices (whole brain) and 284 volumes.

#### Analysis of the fMRI data

Raw DICOM images were converted to NIfTI format using the software MRIConvert (2.1.0). The fMRI data preprocessing was performed with the Statistical Parametric Mapping (SPM 12, Welcome Department of Imaging Neuroscience, London, UK) software package implemented in the Matlab environment. Following slice-timing correction, functional images were realigned to the first volume by affine registration using the standard, six parameters rigid body spatial transformation method. EPI volumes were then co-registered to the 1 mm isotropic T_1_ anatomical image, which had been previously reoriented to the anterior commissure. EPI volumes were spatially smoothed using Gaussian kernel of 6 mm full width at half maximum.

Following preprocessing, statistical analysis was performed at the single-subject level in the framework of the general linear model. Voxels were identified as significant if p < 0.05 (family-wise error corrected for multiple comparisons on the voxel level). We used this parametric map to position our TMS coil in the motor threshold hunting session.

### Neuronavigated (r)TMS

We used neuronavigated (r)TMS in the main experiment in the motor threshold hunting session and 2) in the subsequent rTMS-EEG sessions. In both, i.e., main and control, experiments, we used a MagPro X100 stimulator (MagVenture, Denmark) with a standard figure-eight coil (MC-B70) to deliver biphasic single and repetitive TMS pulses with the normal coil current direction (280 µs pulse width). During stimulation, the participants sat in a fixed chair equipped with an armrest and their eyes open. We used an in-house built, light aluminum frame structure, equipped with soft-cushioned bilateral head fixation pads and chin rest. We mounted the TMS coil on a variable friction arm (Model 244N, Manfrotto, Italy) and adjusted the position of each element individually to achieve the required comfort of the participant and precise TMS targeting in the spTMS and rTMS sessions.

To accurately guide the TMS coil over the anatomical target in the main experiment, we used a frameless, stereotactic MRI-based real-time neuronavigation system (Brainsight TMS Navigation, Rogue Resolutions Ltd) and coupled it with a Polaris Vicra infrared camera (NDI, Waterloo, Canada). In the motor threshold determination session, the target of spTMS was the motor cortex representation of the first dorsal interosseous muscle, which we had previously identified as the highest of the fMRI local activation maximum derived from the parametric t-map at the anatomical hand knob formation.

In the rTMS-EEG sessions (both experiments), the target location was at the PO3 electrode. In the main experiment, we employed neuronavigation for real-time monitoring of the coil location over the target (within 2 mm) and for recording the coil location, which permitted consistent targeting in subsequent rTMS-EEG sessions. To achieve optimal targeting, we did not completely fix the position of the adjustable coil support, which enabled the investigator to make a small adjustment to the coil position in real-time.

### Determination of motor threshold

In the main experiment, we recorded the surface electromyogram from the right first dorsal interosseous muscle with an Ag–AgCl electrode pair in a belly-tendon montage. Raw signals (sampling rate 5 kHz) were amplified, band-pass filtered between 2 Hz and 3 kHz and digitized with a micro 1,401 AD converter (Cambridge Electronic Design, Cambridge, UK). Data collection was controlled by Signal Software (Cambridge Electronic Design, version 4.08).

The EEG electrode array was already in position during the motor threshold hunting session, in order to keep a constant scalp-coil distance in the motor threshold hunting and the subsequent rTMS-EEG sessions. This was a necessary step because the TMS-induced magnetic field (and hence the induced EF) changes as the inverse cube of the distance. No simultaneous EEG recordings were performed during the motor threshold hunting session.

We searched for the anatomical hot spot for the target muscle by initially positioning the coil over the scalp projection area of the fMRI peak voxel with the highest statistical t-value using the neuronavigation system. We then determined the optimal coil position (orientation, angle) in which spTMS elicited the strongest motor evoked potentials (MEP) in the target muscle.

Following optimal positioning, we estimated the RMT by determining the minimum stimulation intensity, expressed as a percentage of MSO, with which at least three out of six TMS pulses produced MEPs with a $\ge 50\;\upmu V$ peak-to-peak amplitude in the resting target muscle^51^. Motor threshold hunting started with an intensity of 30% of MSO that was gradually increased in 5% steps until spTMS consistently evoked MEPs with $\ge 50\; \upmu V$ amplitude. Thereafter, we reduced the stimulation intensity in 1% steps until the RMT was estimated. During the entire procedure, we encouraged the participants to indicate the presence of perceivable effects, such as discomfort due to cranial muscle activation or dizziness, and to inform the investigator if the stimulation was not tolerable. Because of tolerability issues, we set a limit for the stimulation intensity at 75% of MSO. If RMT was not detected by then, it was labeled “undefined” (n = 3). The RMT (n = 14) was on average 54.79% ± 12.29% (SD) MSO.

### Head modeling and EF calculations

We performed individual high-resolution, anatomically realistic head modeling and EF calculations using the Simulation of Non-invasive Brain Stimulation (SimNIBS) software package^52^. These calculations were performed twice: The first time to determine the dose for rTMS (at the single-subject level), and the second time to retrospectively estimate the spatial characteristics and magnitude of the rTMS-induced EF at the group-level. We used the SimNIBS versions 2.0.1 and 2.1.2 for the prospective and retrospective EF calculations, respectively. For both calculations, we set the scalp-to-coil distance to 11 mm in order to take the EEG electrodes into consideration.

The mri2mesh function automatically generated tetrahedral volume meshes of the head from T_1_- and T_2_-weighted structural MR images^53^. The final head mesh consisted of approx. 3,500,000 tetrahedral elements and five tissue compartments. Table 2 shows the five tissue compartments and their conductivity values. We performed EF calculations using the finite element method and the built-in MC-B70 validated coil.

**Table 2:** The five tissue compartments of the head model and their conductivity values [S/m].

Tissue compartment	Conductivity [S/m]
Scalp	0.465
Skull	0.010
CSF	1.654
GM^a^	0.275
WM^a^	0.126

*CSF* cerebrospinal fluid, *GM* gray matter, *WM* white matter.

^a^Indicate anisotropic conductivity values estimated from diffusion tensors using the volume-normalized approach in the retrospective EF calculations^54^.

The goal of the second, retrospective computation was to compare the resulting EFs of the currently used, near threshold approach to our novel approach. For this, we used the maximum stimulator output data derived from the motor threshold determination and rTMS-EEG sessions. In the retrospective computation, we used improved procedures for creating individual head models by assigning anisotropic values to the gray and white matter compartments. We furthermore performed a region of interest (ROI) analysis by focusing on the EF in the parietal and occipital regions.

We also characterized the magnitude of the absolute EF as well as of its normal component, since there is a current debate about the physiological effects of the spatial components of the EF^35,36^. The normal component of the EF can distinguish the depolarizing inward and hyperpolarizing outward EFs^55^. There are claims that the normal component in the wall of the motor cortex is the physiologically effective constituent in the induction of motor evoked potentials in humans^56^. However, considerable skepticism remains about the physiological efficacy of the normal component of the EF^35,36,56^. For example, the peak values of the absolute EF in the gyral crown of the motor cortex is a similarly plausible component in producing a liminal response in the motor cortex^35^. Given this uncertainty in the TMS literature we analyzed both the absolute EF and its normal component.

In the group-level analysis, we provide the peak (99.9th percentile), median and mean EF values. In the group-level ROI analysis, first, we calculate the mean EF strength for each ROI separately at the single-subject level. Then, we calculate median, mean and 95% CIs at the group-level.

### EEG acquisition

We performed EEG recordings (1) to estimate the individual alpha band peak frequency (IAF) and (2) to characterize the immediate electrophysiological effects and short-lasting aftereffects of rTMS. We attained scalp EEG data with a 24-bit, battery-powered, active channel amplifier with 64 Ag/AgCl active EEG electrodes (actiCAP, BrainVision LLC, Germany) at a 2.5 kHz sampling rate, and without hardware filters (actiChamp, Brain Vision LLC, Germany). Ground and reference electrodes were located at Fpz and FCz, respectively. Impedance values were maintained below 20 kΩ.

### Estimating individual alpha band peak frequencies

In both experiments, we estimated IAF at the beginning of each rTMS-EEG session in order to fine-tune the stimulation frequency in the rhythmic rTMS condition. We recorded two 4-min blocks of continuous, resting state EEG, one block with eyes open and the other with eyes closed. We instructed our participants to sit calmly, stay relaxed, not to move their limbs or face muscles, and try to avoid any repetitive mental activity such as reproducing any texts, lyrics or melodies. Participants wore QuietControl 30 wireless headphones with active noise reduction and proper earbud size during the EEG recordings (Bose Corporation, USA).

We performed offline data analysis with the FieldTrip toolbox for EEG- and MEG analysis (version 20170119, https://fieldtrip.fcdonders.nl)^57^. The data was initially segmented into 2 s epochs with 50% overlap, re-referenced to the common average, detrended, demeaned, high-pass (0.1 Hz) and low-pass (40 Hz) filtered with an infinite impulse response filter type Butterworth. The trials were visually inspected for outliers and were rejected based on variability.

Frequency analysis between 1 and 20 Hz with 0.5 Hz increments was performed with the multitaper frequency transformation (‘mtmfft’) method based on discrete prolate spheroidal sequences. After averaging over trials, we determined peak alpha power (and IAF) in the range from 8 to 12 Hz by visual inspection for both “eyes open” and “eyes closed” condition. However, if we could not determine peak alpha frequency from the eyes open condition, we used the eyes closed condition instead (five cases). The average IAF was 10.3 ± 1.0 Hz.

### Simultaneous rTMS and EEG

In each rTMS-EEG session of the main experiment, we employed rhythmic (main) and arrhythmic (active control) rTMS protocols over the same target location with the same stimulation intensity^12,15^. In the control experiment, we employed a single rhythmic sham rTMS protocol by tilting the coil with 90° angle.

In the rhythmic protocol, we set the pulse repetition frequency at IAF. The stimulation frequency and the number of pulses for the rhythmic rTMS burst were preprogrammed in the TMS device, and the start of each rTMS burst was controlled externally via PsychoPy (version 1.83.01) using a parallel port and a Bayonet Neill–Concelman connector^58,59^.

In the arrhythmic protocol, we set a pseudorandom stimulation frequency, and the delivery of each pulse was controlled externally via PsychoPy. Despite pseudorandomization, rhythmic patterns in the alpha frequency band can still emerge by chance for at least several consecutive rTMS pulses^15^. Therefore, we predefined the timing of the TMS pulses so that frequencies in the alpha frequency band (8–12 Hz) and their harmonics and subharmonics were not allowed to occur between the pulses (for instance 4 and 16 Hz for 8 Hz; 4.5 Hz and 18 Hz for 9 Hz; 5 and 20 Hz for 10 Hz; 5.5 Hz and 22 Hz for 11 Hz; 6 and 24 Hz for 12 Hz).

Each rTMS burst contained 20 pulses and each block contained 25 rTMS bursts. The interburst interval was randomly selected to be 10 s or 11 s. In each rTMS-EEG session, we delivered five rhythmic and five arrhythmic blocks, the order of which was randomized for each session and participant.

In the main experiment, the intensity of the rTMS burst was set based on the desired, prospectively estimated EF strength. In the control experiment, we used a fixed stimulation intensity at 29% of the device output. Apart from the stimulation intensity and the coil angle, all the remaining stimulation parameters were the same in the real rhythmic protocols (main experiment) and the sham rhythmic protocol (control experiment).

All stimulation protocols were performed with the participants at rest and instructed to keep their eyes open. During each block, a white noise, auditory masking stimulus was delivered through the QuietControl 30 wireless earphones. At the beginning of each session, we asked the participants to indicate the maximum sound volume that they could tolerate for more than an hour. The sound volume of the white noise was always kept below the manufacturer’s recommended safety limits. This procedure minimized but did not completely eliminate the participants’ ability to hear the click sound produced by the discharge of the coil^60,61^.

### Analysis of rTMS-EEG

#### EEG preprocessing

We performed offline data analysis using the FieldTrip toolbox (version 20180114, https://fieldtrip.fcdonders.nl)﻿^57^ with a custom-made MATLAB code. We first segmented the EEG data into trials of 8.5 s length that were time-locked to the offset of the rTMS burst in an interval from 3.5 s before to 5 s after the last TMS pulse. In the main experiment, each session contained ten blocks of both rhythmic and arrhythmic stimulation. We appended all blocks from a session into one data file and performed the preprocessing blinded to the stimulation type. Each dataset contained 125 trials for the rhythmic and 125 for the arrhythmic stimulation condition. The sham stimulation session contained 125 trials. All the following steps were identical in the main and control experiment.

We defined a ringing artifact of the TMS pulse as the time interval from 4 ms before to 9 ms after the pulse and excised them from the data. As the data still contained residual decay artifacts we ran an independent component analysis (fastICA) with 63 components. To define which components corresponded to decay artifacts we averaged the components’ signal 50 ms after TMS pulse over all trials. We rejected components whose amplitude exceeded 30 µV. On average, 0.5 ± 1.2 (Low); 1.2 ± 1.2 (Medium); 1.3 ± 1.2 (High) components were defined as decay artifacts. The time intervals around the TMS pulse were replaced via Piecewise Cubic Hermite Interpolation (pchip).

The data then was re-referenced to the common average and was down-sampled to 1,250 Hz. We inspected the data to determine which channels or trials contained artifacts using a semi-automatic algorithm adapted from ARTIST^23^ that contained three main criteria. First, we estimated the power of each trial. The outlier power values for each trial and channel were defined as elements lying more than 1.5 interquartile ranges above the upper quartile or below the lower quartile. We marked trials or channels that corresponded to outlier values as ‘bad’. If the number of ‘bad’ channels was less than 20% of the total amount, we interpolated the channels only within the ‘bad’ trial using the weighted signal of neighbor channels. If the percentage was higher, the trial was rejected. We then removed channels with a large standard deviation (STD > 30 µV), which was related to channel movements under the TMS coil. In the next step, we defined channels with line noise or high impedance values. For that, we estimated the correlation coefficient of the signal from a channel with the signal of its neighbors. We removed channels with a low correlation coefficient (CorrCoef < 0.4). On average, 4.2 ± 1.8 (Low), 3.8 ± 1.9 (Medium), 3.8 ± 2.1 (High) channels were interpolated using the weighted signal from neighbor channels.

As the last preprocessing step, we defined eye-related artifacts via the second ICA run. We reduced the number of independent components due to interpolated channels. Components corresponding to blinks, saccades and other eye movements were rejected. On average, we rejected 3.1 ± 1.8 (Low), 2.9 ± 1.3 (Medium), and 2.8 ± 1.2 (High) components during the second ICA run. For the further analysis we used 108.4 ± 7.4 (Low), 107.6 ± 5.1 (Medium) and 106.9 ± 9.4 (High) trials with rhythmic and 107.3 ± 7.1 (Low), 107.9 ± 8.6 (Medium), and 109.0 ± 7.4 (High) trials with arrhythmic stimulation.

#### Phase locking value

We chose the PLV to describe the degree of synchronization of the EEG signal by an external repetitive force. PLV is based on the measurement of phase alignment of the signal to external pulses or stimulated channels^20^.

First, we simulated the sinusoidal wave as an additional channel for each trial at the individual alpha frequency. The phase of the simulated wave was aligned to the TMS offset. The data was decomposed by complex Morlet wavelet. The wavelets contained five cycles with a three-Gaussian window. The decomposition was performed for the trial interval from 3.5 before to 2.5 s after TMS offset.

Then, we computed PLV between the phase component of the simulated wave with the remaining channels as follow:

$$PLV = \frac{1}{N}\left|{\sum }_{n=1}^{N}{e}^{i({\Phi }_{ong}(n)-{\Phi }_{IAF}(n))}\right|,$$

where N is the number of repetitions (trials), Φ_ong_(n) and Φ_IAF_(n) are the instantaneous phase values at the time points n of the ongoing EEG signal and simulated sinusoidal wave at IAF, respectively. The values range from 0 to 1. The PLVs were baseline-normalized by dividing the values at each sample by the value average during the 3.0 to 2.5 s prior to TMS offset (In Fieldtrip: relative normalization).

#### Statistical analysis

Differences in PLVs between rhythmic, arrhythmic and sham stimulation conditions were subjected to cluster-based permutation statistical analysis (two-tailed) at the respective stimulation frequency (8–12 Hz). In the main experiment, we used the dependent t-test between to compare the rhythmic and arrhythmic conditions at the Low, Medium and High sessions separately. In addition, we used the independent t-test to compare the real rhythmic conditions with the control (sham) condition. To control for multiple comparisons, we applied a non-parametric randomization approach. This procedure uses 1,000 randomizations to estimate the probability that a given number of significant electrodes (p < 0.05) can be expected by chance.

#### Control analyses

In order to ensure that the steps used in the rTMS-EEG data analysis cannot account for the observed pattern of findings we performed an additional control experiment by stimulating a piece of meat (chicken, phantom). Moreover, we used our control dataset consisting of artifact-free resting state EEG data to study whether our preprocessing pipeline can spuriously induce the degree of neural synchronization. For both analyses, we used the identical pipeline for data preprocessing and PLV analysis, as described for the main analysis.

In the phantom experiment, rhythmic rTMS was applied at the highest intensity used in the main group and sham group—29% of maximum stimulator output. Pulses were applied in five blocks at stimulation frequency 10 Hz. The data were recorded from 32 EEG channels.

The control dataset was taken from EEG data recorded from 16 participants before each rTMS-EEG session with the initial aim of estimating the individual alpha frequencies (IAF). The data contain a realistic amount of noise with realistic time–frequency characteristics. Because we applied no rTMS during resting state EEG data recordings, we simulated TMS pulses by periodically removing data segments and interpolating them using the IAF.

Data segments of lengths comparable to the main dataset were marked with arbitrary ‘TMS pulse’ onsets based on IAF. The three sessions from each participant were appended to one dataset in order to increase the number of trials. An average of 104.2 ± 6.5 trials were used for the analysis. This is comparable with the trial numbers used in the main analysis (for example, 108.4 ± 7.4 for low intensity).

## Supplementary information

Supplementary information.

## References

[1] Buzsáki G, Logothetis N, Singer W (2013). Scaling brain size, keeping timing: Evolutionary preservation of brain rhythms. Neuron.

[2] Watson BO, Buzsáki G (2015). Sleep, memory & brain rhythms. Daedalus.

[3] Rabiller G, He JW, Nishijima Y, Wong A, Liu J (2015). Perturbation of brain oscillations after ischemic stroke: A potential biomarker for post-stroke function and therapy. Int. J. Mol. Sci..

[4] Anastassiou CA, Koch C (2015). Ephaptic coupling to endogenous electric field activity: Why bother?. Curr. Opin. Neurobiol..

[5] Bergmann TO, Karabanov A, Hartwigsen G, Thielscher A, Siebner HR (2016). Combining non-invasive transcranial brain stimulation with neuroimaging and electrophysiology: Current approaches and future perspectives. Neuroimage.

[6] Fröhlich F (2015). Experiments and models of cortical oscillations as a target for noninvasive brain stimulation. Prog. Brain Res..

[7] Paulus W, Peterchev AV, Ridding M (2013). Transcranial electric and magnetic stimulation: Technique and paradigms. Handb. Clin. Neurol..

[8] Gomez-Tames J, Hamasaka A, Laakso I, Hirata A, Ugawa Y (2018). Atlas of optimal coil orientation and position for TMS: A computational study. Brain Stimul..

[9] Liu, A. *et al.* Immediate neurophysiological effects of transcranial electrical stimulation. *Nat. Commun.* **9**, 5092 (2018). 10.1038/s41467-018-07233-7 PMC6269428 30504921

[10] Opitz A (2015). Is sham cTBS real cTBS? The effect on EEG dynamics. Front. Hum. Neurosci..

[11] Sahlsten H (2015). Electric field navigated transcranial magnetic stimulation for chronic tinnitus: A pilot study. Int. J. Audiol..

[12] Thut G (2011). Rhythmic TMS causes local entrainment of natural oscillatory signatures. Curr. Biol..

[13] Hanslmayr S, Matuschek J, Fellner MC (2014). Entrainment of prefrontal beta oscillations induces an endogenous echo and impairs memory formation. Curr. Biol..

[14] Romei V (2016). Causal evidence that intrinsic beta-frequency is relevant for enhanced signal propagation in the motor system as shown through rhythmic TMS. Neuroimage.

[15] Albouy P, Weiss A, Baillet S, Zatorre RJ (2017). Selective entrainment of theta oscillations in the dorsal stream causally enhances auditory working memory performance. Neuron.

[16] Krause MR, Vieira PG, Csorba BA, Pilly PK, Pack CC (2019). Transcranial alternating current stimulation entrains single-neuron activity in the primate brain. Proc. Natl. Acad. Sci..

[17] Ozen S (2010). Transcranial electric stimulation entrains cortical neuronal populations in rats. J. Neurosci..

[18] Haegens S, Cousijn H, Wallis G, Harrison PJ, Nobre AC (2014). Inter- and intra-individual variability in alpha peak frequency. Neuroimage.

[19] Thut G, Schyns PG, Gross J (2011). Entrainment of perceptually relevant brain oscillations by non-invasive rhythmic stimulation of the human brain. Front. Psychol..

[20] Lachaux JP, Rodriguez E, Martinerie J, Varela FJ (1999). Measuring phase synchrony in brain signals. Hum. Brain Mapp..

[21] Glass L (2001). Synchronization and rhythmic processes in physiology. Nature.

[22] Romei V, Gross J, Thut G (2012). Report sounds reset rhythms of visual cortex and corresponding human visual perception. Curr. Biol..

[23] Wu W (2018). ARTIST: A fully automated artifact rejection algorithm for single-pulse TMS-EEG data. Hum. Brain Mapp..

[24] Yuan H, Shou G, Gleghorn D, Ding L, Cha Y (2017). Resting state functional connectivity signature of treatment effects of repetitive transcranial magnetic stimulation in Mal de Debarquement syndrome. Brain Connect..

[25] Rosanova M (2009). Natural frequencies of human corticothalamic circuits. J. Neurosci..

[26] Herring JD, Thut G, Jensen O, Bergmann TO (2015). Attention modulates TMS-locked alpha oscillations in the visual cortex. J. Neurosci..

[27] Vöröslakos M (2018). Direct effects of transcranial electric stimulation on brain circuits in rats and humans. Nat. Commun..

[28] Peterchev AV, Goetz SM, Westin GG, Luber B, Lisanby SH (2013). Pulse width dependence of motor threshold and input–output curve characterized with controllable pulse parameter transcranial magnetic stimulation. Clin. Neurophysiol..

[29] Hallett M (2007). Transcranial magnetic stimulation: A primer. Neuron.

[30] Rossi S, Hallett M, Rossini PM, Pascual-Leone A, Safety of TMS Consensus Group, T. S. of T. C (2009). Safety, ethical considerations, and application guidelines for the use of transcranial magnetic stimulation in clinical practice and research. Clin. Neurophysiol..

[31] Di Lazzaro V, Oliviero A, Pilato F, Saturno E, Dileone M (2004). The physiological basis of transcranial motor cortex stimulation in conscious humans. Clin. Neurophysiol..

[32] Epstein CM (1999). Localization and characterization of speech arrest during transcranial magnetic stimulation. Clin. Neurophysiol..

[33] Berényi A, Belluscio M, Mao D, Buzsáki G (2012). Closed-loop control of epilepsy by transcranial electrical stimulation. Science.

[34] Huang Y, Parra LC (2019). Can transcranial electric stimulation with multiple electrodes reach deep targets?. Brain Stimul..

[35] Bungert A, Antunes A, Espenhahn S, Thielscher A (2017). Where does TMS stimulate the motor cortex? Combining electrophysiological measurements and realistic field estimates to reveal the affected cortex position. Cereb. Cortex.

[36] Laakso I, Murakami T, Hirata A, Ugawa Y (2018). Where and what TMS activates: Experiments and modeling. Brain Stimul..

[37] Laakso I, Mikkonen M, Koyama S, Hirata A, Tanaka S (2019). Can electric fields explain inter- individual variability in transcranial direct current stimulation of the motor cortex?. Sci. Rep..

[38] Ziemann U (2017). Thirty years of transcranial magnetic stimulation: Where do we stand?. Exp. Brain Res..

[39] Huang Y-Z (2017). Plasticity induced by non-invasive transcranial brain stimulation: A position paper. Clin. Neurophysiol..

[40] Lefaucheur JP (2014). Evidence-based guidelines on the therapeutic use of repetitive transcranial magnetic stimulation (rTMS). Clin. Neurophysiol..

[41] Nielsen JD (2018). Automatic skull segmentation from MR images for realistic volume conductor models of the head: Assessment of the state-of-the-art. Neuroimage.

[42] Vorwerk J, Oostenveld R, Piastra MC, Magyari L, Wolters CH (2018). The FieldTrip-SimBio pipeline for EEG forward solutions. Biomed. Eng. Online.

[43] Saturnino GB, Thielscher A, Madsen KH, Kn TR, Weise K (2019). A principled approach to conductivity uncertainty analysis in electric field calculations. Neuroimage.

[44] Rimpil V, Koulouri A, Lucka F, Kaipio JP, Wolters CH (2019). Improved EEG source localization with Bayesian uncertainty modelling of unknown skull conductivity. Neuroimage.

[45] Moliadze V, Atalay D, Antal A, Paulus W (2012). Close to threshold transcranial electrical stimulation preferentially activates inhibitory networks before switching to excitation with higher intensities. Brain Stimul..

[46] Lenz M, Galanis C, Müller-Dahlhaus F, Opitz A, Wierenga CJ,  Szabó G,  Ziemann U, Deller T, Funke K, Vlachos A (2016). Repetitive magnetic stimulation induces plasticity of inhibitory synapses. Nat. Commun..

[47] Klimesch W, Sauseng P, Hanslmayr S (2007). EEG alpha oscillations: The inhibition-timing hypothesis. Brain Res. Rev..

[48] Mazaheri A, Jensen O (2008). Asymmetric amplitude modulations of brain oscillations generate slow evoked responses. J. Neurosci..

[49] Romei V, Thut G, Mok RM, Schyns PG, Driver J (2012). Causal implication by rhythmic transcranial magnetic stimulation of alpha frequency in feature-based local vs. global attention. Eur. J. Neurosci..

[50] Oldfield RC (1971). The assessment and analysis of handedness: The Edinburgh inventory. Neuropsychologia.

[51] Conforto AB, Z’Graggen W, Kohl AS, Rösler KM, Kaelin-Lang A (2004). Impact of coil position and electrophysiological monitoring on determination of motor thresholds to transcranial magnetic stimulation. Clin. Neurophysiol..

[52] Thielscher, A., Antunes, A. & Saturnino, G. B. Field modeling for transcranial magnetic stimulation: A useful tool to understand the physiological effects of TMS? In *Proc. Annu. Int. Conf. IEEE Eng. Med. Biol. Soc. EMBS* 222–225 (2015) 10.1109/EMBC.2015.7318340 10.1109/EMBC.2015.7318340 26736240

[53] Windhoff M, Opitz A, Thielscher A (2013). Electric field calculations in brain stimulation based on finite elements: An optimized processing pipeline for the generation and usage of accurate individual head models. Hum. Brain Mapp..

[54] Opitz A, Windhoff M, Heidemann RM, Turner R, Thielscher A (2011). How the brain tissue shapes the electric field induced by transcranial magnetic stimulation. Neuroimage.

[55] Rahman A (2013). Cellular effects of acute direct current stimulation: somatic and synaptic terminal effects. J. Physiol..

[56] Fox PT (2004). Column-based model of electric field excitation of cerebral cortex. Hum. Brain Mapp..

[57] Oostenveld R,  Fries P, Maris E, Schoffelen JM (2011). FieldTrip: Open source software for advanced analysis of MEG, EEG, and invasive electrophysiological data. Comput. Intell. Neurosci..

[58] Peirce JW (2007). PsychoPy-psychophysics software in python. J. Neurosci. Methods.

[59] Peirce JW (2009). Generating stimuli for neuroscience using PsychoPy. Front. Neuroinform..

[60] Conde V (2019). The non-transcranial TMS-evoked potential is an inherent source of ambiguity in TMS-EEG studies. Neuroimage.

[61] Nikulin, V. V., Kicić, D., Kähkönen, S. & Ilmoniemi, R. J. Modulation of electroencephalographic responses to transcranial magnetic stimulation: Evidence for changes in cortical excitability related to movement. *Eur. J. Neurosci.* **18**, 1206–1212 (2003). 10.1046/j.1460-9568.2003.02858.x 12956719

