# Supplementary material for: Weak rTMS-induced electric fields produce neural entrainment in humans
Source: Sci Rep. 2020 Jul 20;10:11994. doi: 10.1038/s41598-020-68687-8 (PMC7371859; doi:10.1038/s41598-020-68687-8)
Supplement: Supplementary file 1 — Supplementary information. [file 41598_2020_68687_MOESM1_ESM.pdf]

Weak rTMS-induced electric fields produce neural entrainment in humans

Elina Zmeykina[1], Matthias Mittner[2], Walter Paulus[1] and Zsolt Turi*[1]

[1]Department of Clinical Neurophysiology, University Medical Center Goettingen, Göttingen, Germany

[2]Department of Psychology, University of Tromsø, Norway

[3]Department of Neuroanatomy, Institute of Anatomy and Cell Biology, University of Freiburg, Germany

*Correspondence: zsolt.turi@med.uni-goettingen.de

Supplemental Method

Validation measurement

Since rTMS devices typically operate at high stimulation intensities (≥ 30% MSO) we first validated the output stability of our MagPro X100 stimulator with the MC-B70 coil in the lower stimulation intensity regimen. For that purpose, we measured the induced EF waveforms and peak voltage values with an external induction coil, which was connected to a digital storage oscilloscope (Rigol DS1052E). The tests were performed at intensities from five to 20 percent of MSO with 1% increments and at 30% of MSO. We applied 20 TMS pulses at each pre-determined stimulation intensity. The stimulator produced detectable and stable TMS pulses starting at 8% of MSO. This prospective validation measurement determined the practical lower limit for the rTMS dose, and we ensured that all participants received a stimulation intensity of ≥ 8% MSO. This value corresponds to the induced peak value of an absolute EF strength of ca. 20 $^{mV}/_{mm}$. Note that TMS pulses weaker than 8% MSO probably produced electromagnetic-fields but our external induction coil was unable to detect them.

Supplemental Results

Index	PMID	Article	Method	Threshold percent	MSO
1	28343866	Albouy et al. [1]	FXD	i/r	60
2	29247630	Ando et al. [2]	RMT	90	n/r
3	27812319	Bai et al. [3]	RMT	90	n/r
4	28928648	Bharath et al. [4]	RMT	90	n/r
5	29241839	Cao et al. [5]	RMT	100	44.5
6	27600845	Capotosto et al. [6]	RMT	100	n/r
7	30099627	Cha et al. [7]	RMT	110	n/r
8	29060275	Chen et al. [8]	RMT	110	n/r
9	27445730	D'Agata et al. [9]	RMT	80	n/r
10	27215619	Daltrozzo et al. [10]	RMT	90	n/r
11	26679060	DelFelice et al. [11]	RMT	100	58.6/61.9
12	30253222	DiGiacomo et al. [12]	RMT	80	47.4
13	27626224	Emrich et al. [13]	RMT	110	72
14	29984172	Fisher et al. [14]	RMT	90	n/r
15	26608023	Gongora et al. [15]	RMT	80	47.4
16	29770146	He et al. [16]	RMT	100	n/r
17	29224411	Hunter et al. [17]	RMT	80-120	n/r
18	29238296	Jin et al. [18]	RMT	90	n/r
19	26778629	Kamp et al. [19]	RMT	110	n/r
20	28413707	Karton et al. [20]	vMT	80	n/r
21	27138833	Kazemi et al. [21]	RMT	100/120	n/r
22	30233346	Kazemi et al. [22]	RMT	100/120	n/r
23	30386222	Keuper et al. [23]	FXD	i/r	50
24	27909453	Kim et al. [24]	vMT	110	n/r
25	27852164	Kito et al. [25]	MT	120	n/r
26	29277405	Koch et al. [26]	RMT	110	60.8
27	25165064	Li et al. [27]	MT	100	n/r
28	28959194	Li et al. [28]	RMT	110	n/r
29	28614399	Li et al. [29]	RMT	110	n/r
30	29742385	Lowe et al. [30]	RMT	80	52/53
31	28689295	Lozeron et al. [31]	RMT	80	n/r
32	28008080	Moebius et al. [32]	RMT	110	n/r
33	30219485	Nathou et al. [33]	RMT	80	n/r
34	27516735	Nicolo et al. [34]	vMT	90	n/r

35	28160748	Noda et al. [25]	RMT	95	82.2
36	30318052	Noda et al. [35]	RMT	95	78
37	26873935	Oshima [36]	RMT	90	n/r
38	30290037	Prashad et al.[37]	vMT	80	n/r
39	29914282	Rocha et al. [38]	RMT	80	46.2
40	26584867	Romei [39]	AMT	90	41.9
41	27687560	Rousseau [40]	RMT	120	n/r
42	30425640	Shalbaf et al. [41]	vMT	120	n/r
43	29249371	Shields et al. [42]	vMT	90	n/r
44	28539601	Spadone et al. [43]	RMT	100	n/r
45	27428476	Tikka et al. [44]	RMT	80	n/r
46	30295684	Valiulis et al. [45]	MT	100	n/r
47	28902713	Xia et al. [46]	RMT	90	n/r

Table S1. The vast majority of rTMS studies determine stimulation intensity with the so-called near threshold approach.

We performed a systematic search on PubMed of literature published between 2016 and 2018 with the searching terms “rTMS AND EEG” and “rhythmic TMS AND EEG”. Of the 134 hits, we found 47 eligible articles. All published studies had determined the stimulation frequency with the fixed intensity or the motor threshold approach. The articles are ordered alphabetically. Abbreviations: FXD: fixed intensity; i/r: irrelevant; MSO: maximum stimulator output expressed in percentages; MT: motor threshold; n/r: information is not reported; PMID: PubMed identification number; RMT: resting motor threshold; vMT: visually identified motor threshold.

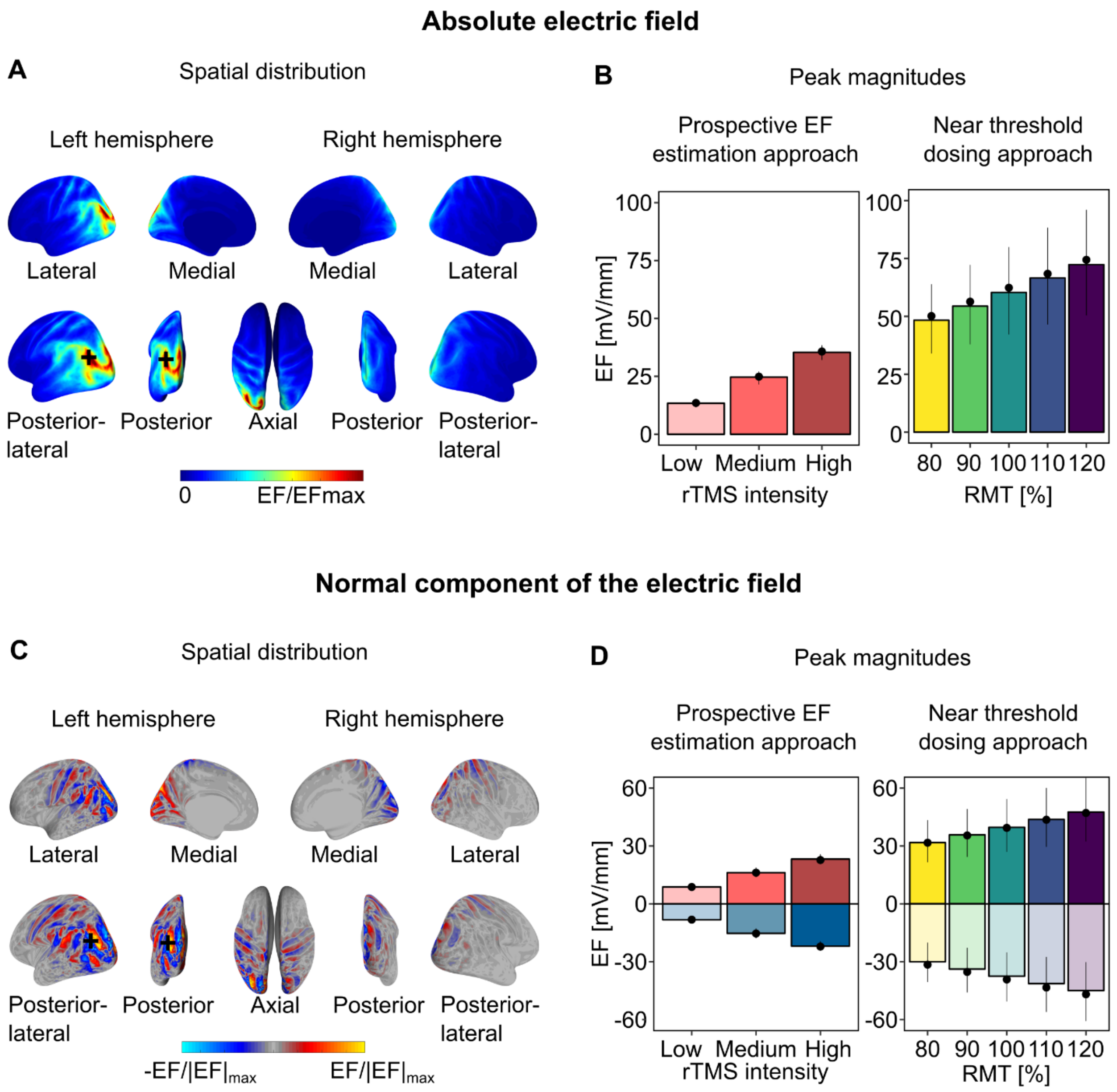

Figure S1. Group-level (n=16) spatial distribution of electric field values.

(A) Absolute electric field values were extracted from the gray matter and projected onto the inflated Freesurfer average template brain. (B) Group-level peak magnitudes of the absolute electric field values in the two intensity selection approaches. (C) Group-level normal component of the electric field values was extracted from the gray matter and

projected onto the inflated Freesurfer average template brain. (D) Peak magnitudes of the normal component of the electric field in the two intensity selection approaches. Peak magnitudes correspond to the 99.9th percentile. A black plus sign shows the positioning of the TMS coil over the PO3 electrode. Bar plots show the mean and dot plots show the median values. Range plots correspond to the 2.5th and the 97.5th percentiles, respectively. Abbreviations: EF – electric field; RMT – resting motor threshold.

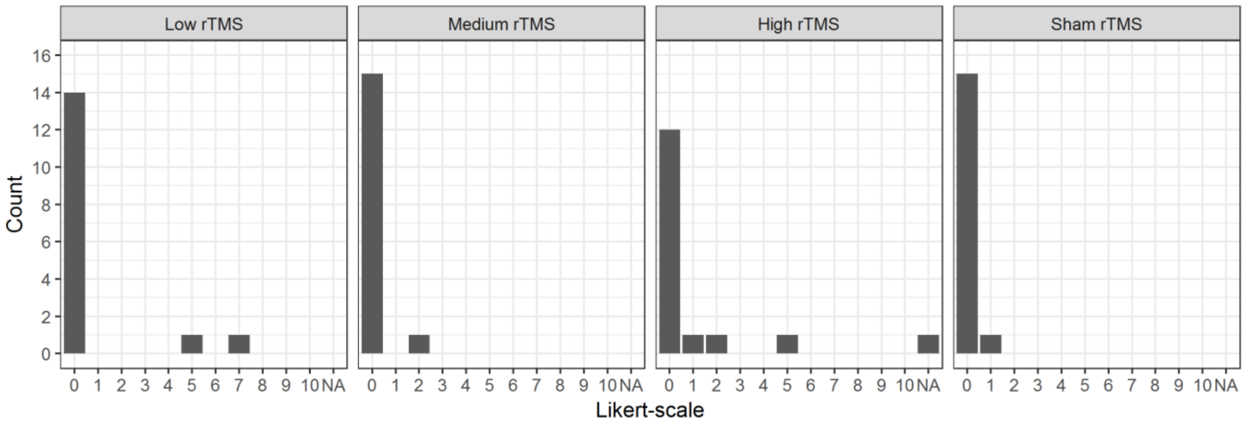

Figure S2. Participants reported a minimal amount of somatosensory perceptual adverse effects.

Both in the main (low, medium and high rTMS) and control experiment (sham rTMS), the participants filled out a post-experimental questionnaire about the somatosensory perceptual adverse effects. Likert scale ranges from 0 and 10, where 0 refers to no sensation detected, 1 indicates minimally detectable sensation and 10 refers to unbearably uncomfortable sensation. NA refers to the case when no answer was provided by the participant.

Supplemental References

1. Albouy, P., Weiss, A., Baillet, S. & Zatorre, R. J. Selective Entrainment of Theta Oscillations in the Dorsal Stream Causally Enhances Auditory Working Memory Performance. *Neuron* **94**, 193-206.e5 (2017).
2. Ando, A. *et al.* Effects of repetitive transcranial magnetic stimulation (rTMS) on attribution of movement to ambiguous stimuli and EEG mu suppression. *Brain Res.* **1680**, 69–76 (2018).
3. Bai, Y. *et al.* Evaluating the Effect of Repetitive Transcranial Magnetic Stimulation on Disorders of Consciousness by Using. *Front. Neurosci.* **10**, 437 (2016).
4. Bharath, R. D. *et al.* A Single Session of rTMS Enhances Small-Worldness in Writer ' s Cramp : Evidence from Simultaneous. *Front. Hum. Neurosci.* **11**, 443 (2017).
5. Cao, D., Li, Y., Niznikiewicz, M. A. & Tang, Y. The theta burst transcranial magnetic stimulation over the right PFC a ff ects electroencephalogram oscillation during emotional processing. *Prog. Neuropsychopharmacol. Biol. Psychiatry* **82**, 21–30 (2018).
6. Capotosto, P. *et al.* Task and Regions Speci fi c Top-Down Modulation of Alpha Rhythms in Parietal Cortex. *Cereb. Cortex* **27**, 4815–4822 (2017).
7. Cha, Y. H. *et al.* Electrophysiological Signatures of Intrinsic Functional Connectivity Related to rTMS Treatment for Mal de Debarquement Syndrome. *Brain Topogr.* **31**, 1047–1058 (2018).
8. Chen, Y. *et al.* Assessing rTMS Effects in MdDS : Cross - modal Comparison between Resting State EEG and fMRI Connectivity. *Conf. Proc. 39th Annu. Int. Conf. IEEE Eng. Med. Biol. Soc.* 1950–1953 (2017)

doi:10.1109/EMBC.2017.8037231.

9. D'Agata, F. *et al.* Cognitive and Neurophysiological Effects of Non-invasive Brain Stimulation in Stroke Patients after Motor Rehabilitation. *Front. Behav. Neurosci.* **10**, 135 (2016).

10. Daltrozzo, J., Kotchoubey, B., Gueler, F. & Karim, A. A. Effects of Transcranial Magnetic Stimulation on Body Perception : No Evidence for Specificity of the Right Temporo-Parietal Junction. *Brain Topogr.* **29**, 704–715 (2016).

11. Del Felice, A. *et al.* Neurophysiological , psychological and behavioural correlates of rTMS treatment in alcohol dependence. *Drug Alcohol Depend.* **158**, 147–153 (2016).

12. Di Giacomo, J. *et al.* Neuroscience Letters Repetitive Transcranial Magnetic Stimulation changes absolute theta power during cognitive / motor tasks. *Neurosci. Lett.* **687**, 77–81 (2018).

13. Emrich, S. M., Johnson, J. S., Sutterer, D. W. & Postle, B. R. Comparing the Effects of 10-Hz Repetitive TMS on Tasks of Visual STM and Attention. *J. Cogn. Neurosci.* **29**, 286–97 (2017).

14. Fisher, R. *et al.* Epilepsy & Behavior Case Reports Repetitive transcranial magnetic stimulation directed to a seizure focus localized by high-density EEG : A case report. *Epilepsy Behav. Case Reports* **10**, 47–53 (2018).

15. Gongora, M. *et al.* Low-frequency rTMS over the Parieto – frontal network during a sensorimotor task : The role of absolute beta power in the sensorimotor integration. *Neurosci. Lett.* **611**, 1–5 (2016).

16. He, F. *et al.* Clinical Study Effects of 20Hz Repetitive Transcranial Magnetic Stimulation on Disorders of Consciousness : A Resting-State

Electroencephalography Study. *Neural Plast.* 5036184 (2018).

17. Hunter, A. M. *et al.* Change in Quantitative EEG Theta Cordance as a Potential Predictor of Repetitive Transcranial Magnetic Stimulation Clinical Outcome in Major Depressive Disorder. *Clin. EEG Neurosci. EEG Neurosci* **49**, 306–315 (2018).
18. Jin, J.-N. *et al.* The Effects of rTMS Combined with Motor Training on Functional Connectivity in Alpha Frequency Band. *Front. Behav. Neurosci.* **11**, 234 (2017).
19. Kamp, D. *et al.* High frequency repetitive transcranial magnetic stimulation (rTMS) reduces EEG-hypofrontality in patients with schizophrenia. *Psychiatry Res.* **236**, 199–201 (2016).
20. Karton, I. & Bachmann, T. Disrupting dorsolateral prefrontal cortex by rTMS reduces the P300 based marker of deception. *Brain Behav.* **7**, e00656 (2017).
21. Kazemi, R. *et al.* Electrophysiological correlates of bilateral and unilateral repetitive transcranial magnetic stimulation in patients with bipolar depression. *Psychiatry Res.* **240**, 364–375 (2016).
22. Kazemi, R. *et al.* Bilateral Transcranial Magnetic Stimulation on DLPFC Changes Resting State Networks and Cognitive Function in Patients With Bipolar Depression. *Front. Hum. Neurosci.* **12**, 365 (2018).
23. Keuper, K., Terrighena, E. L., Chan, C. C. H. & Junghoefer, M. How the Dorsolateral Prefrontal Cortex Controls Affective Processing in Absence of Visual Awareness – Insights From a Combined EEG-rTMS Study. *Front. Hum. Neurosci.* **12**, 1–19 (2018).
24. Kim, Y. I., Kim, S. M., Kim, H. & Han, D. H. The Effect of High-Frequency Repetitive Transcranial Magnetic Stimulation on Occupational Stress among

Health Care Workers : A Pilot Study. *Psychiatry Investig.* **13**, 622–629 (2016).

25. Kito, S. *et al.* Transcranial Magnetic Stimulation Modulates Resting EEG Functional Connectivity Between the Left Dorsolateral Prefrontal Cortex and Limbic Regions in Medicated Patients With Treatment-Resistant Depression. *Jounal Neuropsychiatry Clin. Neurosci.* **29**, 155–159 (2017).

26. Koch, G. *et al.* Transcranial magnetic stimulation of the precuneus enhances memory and neural activity in prodromal Alzheimer ' s disease. *Neuroimage* **169**, 302–311 (2018).

27. Li, C.-T. *et al.* Cognition-Modulated Frontal Activity in Prediction and Augmentation of Antidepressant Ef fi cacy : A Randomized Controlled Pilot Study. *Cereb. Cortex* **26**, 202–210 (2016).

28. Li, S. *et al.* Theta and Alpha Oscillations during the Retention Period of Working Memory by rTMS Stimulating the Parietal Lobe. *Front. Behav. Neurosci.* **11**, 170 (2017).

29. Li, Y. *et al.* The effects of high-frequency rTMS over the left DLPFC on cognitive control in young healthy participants. *PLoS One* **12**, e0179430 (2017).

30. Lowe, C. J., Staines, W. R., Manocchio, F. & Hall, P. A. The neurocognitive mechanisms underlying food cravings and snack food consumption . A combined continuous theta burst stimulation (cTBS) and EEG study. *Neuroimage* **177**, 45–58 (2018).

31. Lozeron, P. *et al.* Inhibitory rTMS applied on somatosensory cortex in Wilson ' s disease patients with hand dystonia. *J. Neural Transm.* **124**, 1161–1170 (2017).

32. Möbius, M. *et al.* Repetitive transcranial magnetic stimulation modulates the impact of a negative mood induction. *Soc. Cogn. Affect. Neurosci.* **12**, 526–533

(2017).

33. Nathou, C. *et al.* E ff ects of low- and high-frequency repetitive transcranial magnetic stimulation on long-latency auditory evoked potentials. *Neurosci. Lett.* **686**, 198–204 (2018).

34. Nicolo, P., Fargier, R., Laganaro, M. & Guggisberg, A. G. Neurobiological Correlates of Inhibition of the Right Broca Homolog during New-Word Learning. *Front. Hum. Neurosci.* **10**, 371 (2016).

35. Noda, Y., Zomorrodi, R., Daskalakis, J., Blumberger, D. M. & Nakamura, M. Enhanced theta-gamma coupling associated with hippocampal volume increase following high-frequency left prefrontal repetitive transcranial magnetic stimulation in patients with major depression. *Int. J. Psychophysiol.* **133**, 169–174 (2018).

36. Oshima, H. *et al.* Alteration of Duration Mismatch Negativity Induced by Transcranial Magnetic Stimulation Over the Left Parietal Lobe. *Clin. EEG Neurosci.* **48**, 11–19 (2017).

37. Prashad, S., Dedrick, E. S., To, W. T., Vanneste, S. & Filbey, F. M. Testing the role of the posterior cingulate cortex in processing salient stimuli in cannabis users : an rTMS study. *Eur. J. Neurosci.* 1–13 (2018) doi:10.1111/ejn.14194.

38. Rocha, K. *et al.* Low-frequency rTMS stimulation over superior parietal cortex medially improves time reproduction and increases the right dorsolateral prefrontal cortex predominance. *Int. J. Neurosci.* **129**, 523–533 (2018).

39. Romei, V. *et al.* Causal evidence that intrinsic beta-frequency is relevant for enhanced signal propagation in the motor system as shown through rhythmic TMS. *Neuroimage* **126**, 120–130 (2016).

40. Rousseau, E., Melo-silva, C. A., Gakwaya, S. & Sériès, F. Effects of repetitive

transcranial magnetic stimulation of upper airway muscles during sleep in obstructive sleep apnea patients. *J. Appl. Physiol.* **121**, 1217–1225 (2016).

41. Shalbaf, R. *et al.* Non-linear Entropy Analysis in EEG to Predict Treatment Response to Repetitive Transcranial Magnetic Stimulation in Depression. *Front. Pharmacol.* **9**, 1188 (2018).

42. Shields, J., Devier, D. & Foundas, A. Journal of the Neurological Sciences Unilateral repetitive transcranial magnetic stimulation di ff erentially affects younger and older adults completing a verbal working memory task. *J. Neurol. Sci.* **384**, 15–20 (2018).

43. Spadone, S., Sestieri, C., Baldassarre, A. & Capotosto, P. Temporal dynamics of TMS interference over preparatory alpha activity during semantic decisions. *Scient* **7**, 2372 (2017).

44. Tikka, S. K. *et al.* Safety and Efficacy of Adjunctive Θ Burst Repetitive Transcranial Magnetic Stimulation to Right Inferior Parietal Lobule in Schizophrenia Patients With A Pilot , Exploratory Study. *J. ECT* **33**, 43–51 (2017).

45. Valiulis, V., Gerulskis, G., Dapšys, K. & Valavičiūtė, K. The use of MR - less MNI based neuronavigation for 10 Hz rTMS depression therapy : electrophysiological and clinical implications. *Acta Neurobiol. Exp. (Wars).* **78**, 271–280 (2018).

46. Xia, X. *et al.* Long-lasting repetitive transcranial magnetic stimulation modulates electroencephalography oscillation in patients with disorders of consciousness. *Neuroreport* **28**, 1022–1029 (2017).
